# Ocrelizumab alters the circulating metabolome in people with relapsing–remitting multiple sclerosis

**DOI:** 10.1002/acn3.52167

**Published:** 2024-08-26

**Authors:** Fatemeh Siavoshi, Dimitrios C. Ladakis, Ashley Muller, Bardia Nourbakhsh, Pavan Bhargava

**Affiliations:** ^1^ Department of Neurology Johns Hopkins University School of Medicine Baltimore Maryland USA

## Abstract

**Background:**

Circulating metabolite levels are altered in multiple sclerosis (MS) and are associated with MS severity. However, how metabolic profiles shift following highly efficacious therapies, like ocrelizumab remains unclear.

**Objective:**

Circulating metabolite levels are altered in multiple sclerosis (MS) and are associated with MS severity. However, how metabolic profiles shift following highly efficacious therapies, like ocrelizumab remains unclear. To assess changes in the circulating metabolome produced by ocrelizumab treatment in people with relapsing–remitting MS (RRMS).

**Methods:**

Thirty‐one individuals with RRMS eligible for beginning treatment with ocrelizumab were recruited and followed with demographic, clinical, quality‐of‐life, and global metabolomics data collected at each visit. Modules of highly correlated metabolites were identified using the weighted correlation network analysis approach. Changes in each module's eigenmetabolite values and individual metabolites during the study were evaluated using linear mixed‐effects models.

**Results:**

Patients with a mean age of 40.8 (SD = 10.30) years, and median disease duration of 4.0 (IQR = 8.5) years, were monitored for a median of 3.36 (IQR = 1.43) years. Two out of twelve identified sets of metabolites were altered significantly. The first module mainly contained androgenic and pregnenolone steroids (*p*‐value <0.001, coefficient: −0.10). The second module primarily consisted of several lysophospholipids, arachidonic acid, some endocannabinoids, and monohydroxy fatty acid metabolites (*p*‐value = 0.016, coefficient: −0.12), which its reduction was significantly associated with improvement based on overall disability response score (OR 3.09e‐01, 95% CI: 6.83e‐02, 9.09e‐01, *p*‐value = 3.15E‐02).

**Interpretation:**

In this longitudinal observational study, using a global untargeted metabolomics approach, we showed significant alteration in circulating metabolome in RRMS patients undergoing ocrelizumab treatment. In particular, we observed a significant reduction in metabolites involved in the lysophospholipid pathway, which was associated with patients' improvement.

## Introduction

Multiple sclerosis (MS) is a chronic demyelinating disorder of the central nervous system (CNS) with inflammatory and degenerative components.^1^ The lack of definitive diagnostic and prognostic biomarkers makes it challenging to monitor disease progression and assess treatment efficacy, highlighting the need for additional biomarkers for early diagnosis and precise monitoring of MS.^2^ Metabolomics, a relatively new “omics” field, involves the assessment of levels of small molecules in biological matrices and can reflect changes of the metagenomics, the proteome, the microbiome, and the exposome (environmental factors)—which can provide a better insight in MS pathophysiology.^3^, ^4^ Recent advances in metabolomics research have helped define distinct metabolic alterations in the circulating metabolome of people with MS (PwMS) compared to healthy individuals.^5^, ^6^ Furthermore, significant changes in the metabolome of MS patients following specific treatments have also been identified.^7^, ^8^ Further exploration of the metabolome in PwMS holds the potential to uncover novel insights into the underlying mechanisms of the disease and find diagnostic and prognostic biomarkers.^9^

Ocrelizumab, a humanized monoclonal antibody targeting CD20‐positive B cells, has emerged as a highly effective disease‐modifying therapy for various MS subtypes, demonstrating efficacy in reducing relapse rates and delaying disability progression in individuals with relapsing–remitting and progressive forms of the disease.^10^, ^11^, ^12^, ^13^ While the clinical benefits of ocrelizumab are well‐established, a comprehensive understanding of its impact at the molecular level remains incomplete.

In this study, we aimed to provide a comprehensive analysis of the metabolomic changes that occur in RRMS patients starting ocrelizumab treatment and identify correlations between these changes and disease progression. The identification of the main metabolites influenced by ocrelizumab therapy advances our understanding of its mechanism of action and helps with identifying biomarkers that are indicative of treatment response and disease progression. Moreover, the results of our study can be utilized to develop a targeted assay that would be more feasible to deploy in large‐scale validation studies.

## Materials and Methods

### Participants

Enrolled participants were chosen from a pool already enrolled or willing to participate in the Johns Hopkins Precision Medicine Center Biobanking initiative, which requires the collection of serum and plasma at 6‐monthly intervals. Those who were diagnosed with relapsing–remitting MS based on the 2017 McDonald Criteria,^14^ between the ages of 18 and 75, eligible for starting ocrelizumab treatment as determined by their treating neurologist, able to return for visits to Johns Hopkins MS center every 6 months, and willing to sign the informed consent form were recruited in the study.

Patients were excluded from the study if they had been diagnosed with any other neuroinflammatory or neurodegenerative disorder, had received corticosteroids within 30 days prior to the blood draw, had other significant metabolic comorbidities such as uncontrolled hypothyroidism or diabetes, had previously been treated with Rituximab or other chemotherapy agents, or were likely to switch therapy within the next year.

At baseline, patients completed a visit before starting ocrelizumab and had regular follow‐up visits after initiation. During each study visit, their demographic data and clinical characteristics were collected. Moreover, phlebotomy, and various assessments, including the Expanded Disability Status Scale (EDSS),^15^ processing speed test,^16^, ^17^ Multiple Sclerosis Functional Composite (MSFC), and^18^, ^19^ short form Neuro‐QOL (quality of life)^20^ were performed.

Informed consent was obtained from all study participants. Patients could withdraw from the study at any time. The reasons for withdrawal from the study could include but were not limited to patient request, investigator or sponsor discretion, and non‐compliance by the patient, which is defined as the failure to attend scheduled study visits. The research protocol followed in this study was approved by the Johns Hopkins Institutional Review Board.

### Metabolomics analysis

At the end of the study, plasma samples obtained during each study visit underwent metabolomics analysis conducted by Metabolon Inc. in Durham, NC. The analytical procedure involved the thawing of the stored samples, followed by sample preparation using previously published standardized methods.^18^ Subsequently, the prepared samples were subjected to either gas chromatography or liquid chromatography, followed by mass spectrometry as previously described. The resulting mass spectra were matched with a standard library for compound identification. The relative abundance of 1298 identified metabolites was determined using the area under the curve of the mass spectra.

### Statistical analysis

The concentrations of metabolites were obtained from untargeted metabolomics analysis. All identified metabolites were included in the analysis. Metabolites with missing values over 30% were removed, and the remaining missing metabolite values were imputed using the K‐Nearest Neighbors method (10 neighbors were used for each imputation). Finally, the metabolite values were median‐scaled and log‐transformed for normalization.

To identify metabolic pathways associated with ocrelizumab treatment, we used two different types of pathway‐based analyses; first, we grouped metabolites into pathways (with a minimum of five metabolites) based on related biological functions, ranked the obtained *p*‐values in the linear mixed effect (LME) models of individual metabolites, and performed pathway enrichment analysis using gene set enrichment analysis (GSEA). GSEA was used to detect significantly enriched metabolic pathways over the time of study. To quantify the degree to which a predefined metabolic set is enriched we used normalized enrichment score (NES) which considers variations in metabolic pathway size and metabolomic data characteristics.^19^

In an alternative approach, we used weighted gene‐expression correlation network analysis (WGCNA) to cluster highly correlated metabolites and define modules as branches using a correlation network.^20^ For constructing the network of the WGCNA, the soft‐threshold power was set at 12. The first principal component of the identified modules known as eigen‐metabolite was used in subsequent analyses. Then we utilized the eigen‐metabolite of each module as the dependent variable, the duration from the baseline visit (measured in years) as the independent variable, in the LME model adjusting for age at the beginning of the study, sex, and DMT history, to assess the change in the identified metabolite modules. Considering that over 90% of participants in our study were Caucasian, we did not adjust our models for race and ethnicity. A detailed covariate selection process is described in Supplementary Methods.

To evaluate metabolites' changes over time in response to ocrelizumab treatment, for each metabolite, we utilized an LME model. To ensure consistency and simplify comparisons between the results of the network correlation analysis (models using module eigen‐metabolites) and individual metabolite analysis, we used the same set of covariates across all models using individual metabolites as response variables. We considered the metabolite level as the dependent variable, the duration from the baseline visit (measured in years) as the independent variable, and adjusted the model for age at baseline, sex, and prior history of disease‐modifying therapy.

To assess the change in disability outcome measures, we compared the baseline and final EDSS of patients using the Wilcoxon Signed‐Rank Test. Additionally, we evaluated the change in MSFC components, including the processing speed test, 9‐Hole Peg Test (9HPT), 25‐Foot Walk Test (25FWT), and neuro‐QOL items over time utilizing the LME model adjusted for variables including age at baseline, sex, and DMT history. Due to the exploratory nature of this analysis, adjustments for multiple comparisons were not made.

To determine if the changes in metabolites were associated with changes in disability outcomes, we used logistic regression models with improvement status as a dependent variable and change in each eigen‐metabolite per standard deviation (SD) as a covariate adjusting for age at baseline, sex, years of follow‐up, and DMT history. To validate our results, we categorized patients as improved and unimproved based on two different methods. In the first method, we utilized thresholds from the EDSS‐Plus method to classify improvement. Improvement was defined as at least a 1‐point decrease from baseline in EDSS score with a baseline EDSS score less than or equal to 5.5 or 0.5‐point decrease with a baseline EDSS score over 5.5 or at least 20% improvement from baseline in either T25FW, dominant or non‐dominant hand 9HPT (9HPT‐ND) at the last study visit compared to baseline.^21^ In the second method, we used the Overall Disability Response Score (ODRS) method to define patients' improvement.^22^ To calculate the ODRS at the end of the study, we evaluated the change in four components: EDSS, T25FW, dominant hand 9HPT (9HPT‐D), and 9HPT‐ND from the study baseline. For the EDSS, we used the same improvement or worsening threshold as the previous method. For T25FW, 9HPT‐D, and 9HPT‐ND, we considered a reduction of 15% or more and an increase of 15% or more in time from baseline as improvement and worsening thresholds, respectively. For each component, a change that met the improvement threshold was given a score of +1, while a change that met the worsening threshold received a score of −1. We then added the scores for all four components for each patient, resulting in a total score ranging from +4 to −4. A positive score indicates an overall improvement in disability from the baseline. The same logistic model was performed on individual metabolites to estimate the improvement per SD increase in individual metabolites.

To evaluate the relationship between metabolic pathways and patients' self‐reported quality of life, we did two separate analyses; We analyzed the correlation between the changes in eigen‐metabolites and neuro‐QOL items using partial Spearman's correlation adjusting for patients' age at baseline, years of follow‐up, sex, and DMT history. In addition, we used the logistic regression model with the improvement on each item of the neuro‐QOL questionnaire as a dependent variable, and change in each eigen‐metabolite per SD as an independent variable, adjusting for the same covariates. We used the values of previously determined conditional minimal detectable changes for the items of neuro‐QOL to define improvement.^23^

## Results

### Demographic and clinical characteristics of the study cohort

In this study, 31 patients, of whom 21 were women, were monitored for a median (IQR) of 3.35(2.65–4.08) years. The patients had a mean age of 40.8 ± 10.3 years, median (IQR) disease duration of 4 (1–9.5) years, and a mean BMI of 28.1 ± 6.14 kg/m^2^ at the beginning of the study (Table 1). There were at least three samples per patient, with an average interval of 1 year and 3 months between each sample collection. All samples from all time points (a total of 112 samples) were included in the analysis.

**Table 1:** Clinical characteristics and demographics of included participants.

*Baseline*	
Number of subjects	31
Age, year	
Mean ± SD	40.84 ± 10.34
Min, max	19, 61
Female, *n* (%)	21 (67.74)
Race	
Black or African American *n* (%)	3 (9.67%)
White *n* (%)	28 (90.33%)
BMI, kg/m^2^	
Mean ± SD	28.06 ± 6.13
Min, max	18.03, 45.24
Disease duration, year	
Median (IQR)	4.0 (1–9.5)
Min, max	1, 22
Previously DMT‐treated patients, *n* (%)	22 (70.96)
EDSS, median (IQR)	2 (1.25–3)
*Follow‐up*	
Follow‐up duration, year	
Median (IQR)	3.36 (2.65–4.08)
Min, max	1.50, 5.45
Number of improved patients based on ODRS, *n* (%)	9 (29.03)

BMI, body mass index; DMT, disease‐modifying therapy; IQR, interquartile range; ODRS, overall disability response score; SD, standard deviation.

**Figure 1:**
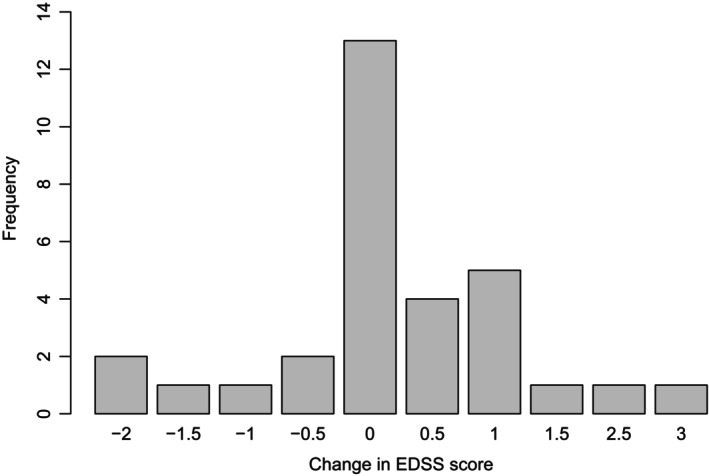
Change in patients' EDSS score during the study.

Compared with pre‐treatment values, EDSS (*p*‐value = 0.291), MSFC components including the processing speed test (*p*‐value = 0.151), 25FWT (*p*‐value = 0.153), 9HPT‐D (*p*‐value = 0.564), and 9HPT‐ND (*p*‐value = 0.707) did not change during the study period and patients were overall stable based on disability outcome measures (Fig. 1). Based on the neuro‐QOL questionnaire, patients experienced an improvement in their overall quality of life. This improvement was reflected in various domains of the questionnaire such as a decrease in the level of stigma (*p*‐value = 0.001) and depression (*p*‐value = 0.030), and an increase in cognitive performance (*p*‐value = 0.018) and satisfaction in their social roles (*p*‐value = 0.039) (Table 2).

**Table 2:** Clinical characteristics and patient‐reported outcomes.

Outcome^a^	Baseline	End of study	*p*‐value^c^
EDSS, median (IQR)^d^	2 (1.25–3)	2 (1.25–4.25)	0.291
Processing speed test^e^	50.48 ± 12.38	51.6 ± 15.31	0.151
Dominant 9HPT^b^, ^d^	28.59 ± 16.16	25.38 ± 10.21	0.564
Non‐dominant 9HPT^b^, ^d^	28.17 ± 11.61	26.04 ± 7.25	0.707
Twenty‐Five FWT^b^, ^d^	6.70 ± 3.50	6.48 ± 3.98	0.153
Neuro‐QOL anxiety^d^	51.93 ± 7.37	49.07 ± 6.05	0.067
Neuro‐QOL cognitive function^e^	45.97 ± 8.04	47.87 ± 7.42	**0.018**
Neuro‐QOL depression^d^	47.42 ± 7.10	45.10 ± 6.29	**0.030**
Neuro‐QOL fatigue^d^	48.49 ± 8.80	46.60 ± 9.29	0.063
Neuro‐QOL ability to participate in social roles^e^	47.07 ± 8.33	48.53 ± 8.99	0.665
Neuro‐QOL satisfaction with social roles^e^	47.99 ± 7.47	48.62 ± 7.19	**0.039**
Neuro‐QOL sleep disturbance^d^	50.30 ± 6.86	48.53 ± 7.96	0.231
Neuro‐QOL stigma^d^	48.84 ± 8.99	45.97 ± 9.11	**0.001**
Neuro‐QOL lower extremity^e^	49.18 ± 9.89	46.37 ± 12.59	0.183
Neuro‐QOL upper extremity^e^	45.77 ± 9.56	47.97 ± 9.47	0.962

^a^ EDSS is presented as the median and interquartile range, while other outcomes are reported as the mean ± standard deviation.

^b^ Measurements for these variables are in seconds.

^c^ *p*‐value calculated using Wilcoxon Signed‐Rank Test for EDSS and using Linear Mixed Effect model for other variables. Values in bold are statistically significant at *p*‐value <0.05.

^d^ An increase in the score of the outcome indicates a deterioration in the patient's condition.

^e^ An increase in the score of the outcome indicates an improvement in the patient's condition.

### Change in the circulating metabolome following ocrelizumab based on individual metabolites and metabolic pathways

In analyses using individual metabolites, we observed significant alterations in various pregnenolone and androgenic steroids, lysophospholipid (LPL) metabolites, benzoate metabolites, pyrimidine metabolites (uracil containing), serine and lactate, after false discovery rate (FDR)‐adjustment (Table 3). The results of changes in all individual metabolites are in the Table S1.

**Table 3:** Metabolites changed significantly following treatment.^a^

Metabolic pathway	Metabolite	Estimate (95% CI)	*p*‐value^b^	FDR
Pregnenolone steroids	Pregnenediol disulfate (C21H34O8S2)	−9.61e‐02 (−1.29e‐01, −6.32e‐02)	1.78E‐07	1.90E‐04
Chemical	Perfluorooctanesulfonate (PFOS)	−1.45e‐01 (−1.99e‐01, −9.08e‐02)	1.17E‐06	6.23E‐04
Benzoate metabolism	Propyl 4‐hydroxybenzoate sulfate	5.93e‐01 (3.46e‐01, 8.39e‐01)	1.02E‐05	3.64E‐03
Pyrimidine metabolism, uracil containing	2′‐deoxyuridine	−6.59e‐02 (−9.41e‐02, −3.77e‐02)	1.64E‐05	4.38E‐03
Pregnenolone steroids	Pregnenetriol disulfate	−7.65e‐02 (−1.12e‐01, −4.14e‐02)	5.29E‐05	1.13E‐02
Pyrimidine metabolism, uracil containing	N‐acetyl‐beta‐alanine	7.86e‐02 (4.19e‐02, 1.15e‐01)	6.85E‐05	1.22E‐02
Androgenic steroids	Androstenediol (3beta,17beta) disulfate (2)	−7.13e‐02 (−1.06e‐01, −3.68e‐02)	1.15E‐04	1.76E‐02
Glycine, serine and threonine metabolism	Serine	−3.95e‐02 (−5.89e‐02, −2.01e‐02)	1.43E‐04	1.92E‐02
Glycolysis, gluconeogenesis, and pyruvate metabolism	Lactate	−5.38e‐02 (−8.07e‐02, −2.69e‐02)	1.87E‐04	2.16E‐02
Chemical	Perfluorooctanoate (PFOA)	−1.11e‐01 (−1.68e‐01, −5.49e‐02)	2.20E‐04	2.16E‐02
Lysophospholipid	1‐linoleoyl‐GPA (18:2)	−9.76e‐02 (−1.47e‐01, −4.79e‐02)	2.39E‐04	2.16E‐02
Secondary bile acid metabolism	Taurocholenate sulfate	−1.02e‐01 (−1.54e‐01, −5e‐02)	2.43E‐04	2.16E‐02

^a^ Significantly changed metabolites ordered by *p*‐value.

^b^ The *p*‐value was calculated using a linear mixed effect model with the metabolite level as the dependent variable and the duration from the baseline visit (measured in years) as the independent variable, adjusted for age at baseline, sex, and prior history of disease‐modifying therapy.

**Figure 2:**
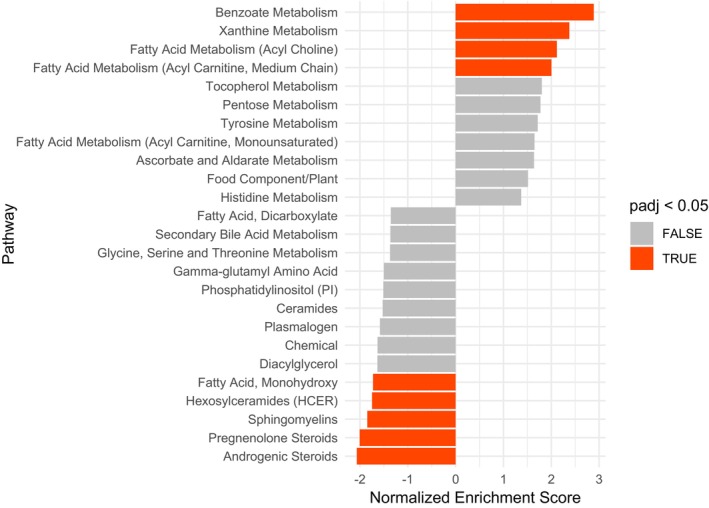
Metabolomics pathway enrichment analysis of metabolic pathways that were altered following ocrelizumab treatment, ranked by the normalized enrichment score (NES). The NES is used to determine the degree to which a metabolic pathway is overrepresented at either the top or bottom of a list of ranked metabolites. A positive score indicates that a metabolic pathway is up‐regulated following treatment, while a negative score indicates that it is down‐regulated. The NES is represented by a bar, with orange indicating significantly altered pathways (Adjusted *p*‐value <0.05) and gray indicating non‐significantly altered ones.

Results were also consistent in pathway‐based analyses. Pathway enrichment analysis, identified benzoate metabolism, androgenic and pregnenolone steroids, xanthine metabolism, sphingomyelins, and fatty acid metabolism (Acyl choline, Acyl carnitine, monohydroxy, and Hexosylceramides) as the most significantly changed metabolic pathways (Fig. 2). Based on WGCNA analysis, metabolites were clustered into 12 modules, of which green and magenta modules were altered significantly following ocrelizumab treatment based on the LME model. The green module mainly contained androgenic and pregnenolone steroids (*p*‐value <0.001, coefficient: −0.10). The magenta module primarily consisted of several LPLs, some endocannabinoid metabolites, monohydroxy fatty acid metabolites, and arachidonic acid (*p*‐value = 0.016, coefficient: −0.12) (Table 4). The complete contents of the magenta and green modules are listed in the Table S2.

**Table 4:** Metabolite in magenta and green modules changed significantly following treatment.

Module	Metabolite	Linear mixed effect model results for individual metabolite change following treatment		
		MM^a^	Estimate (95% CI)	*p*‐value^b^
Magenta	2‐palmitoyl‐GPC (16:0)	0.85	−4.2e‐02 (−7.61e‐02, −7.82e‐03)	1.83E‐02
	2‐hydroxystearate	0.78	−3.06e‐02 (−5.64e‐02, −4.92e‐03)	2.21E‐02
	1‐stearoyl−2‐arachidonoyl‐GPI (18:0/20:4)	0.70	−2.33e‐02 (−3.87e‐02, −7.89e‐03)	4.00E‐03
	N‐stearoyltaurine	0.65	−6.32e‐02 (−1.05e‐01, −2.11e‐02)	4.29E‐03
	Spermidine	0.44	−6.6e‐02 (−1.12e‐01, −2.03e‐02)	5.91E‐03
	Glutamine	0.43	−2.04e‐02 (−3.63e‐02, −4.51e‐03)	1.39E‐02
	2‐arachidonoylglycerol (20:4)	0.38	−6.22e‐02 (−1.16e‐01, −8.76e‐03)	2.52E‐02
	S‐carboxymethyl‐cysteine	0.36	−5.13e‐02 (−9.8e‐02, −4.65e‐03)	3.42E‐02
	2‐hydroxyarachidate	0.34	−7.35e‐02 (−1.22e‐01, −2.5e‐02)	3.94E‐03
Green	Androstenediol (3beta,17beta) disulfate (2)	0.93	−7.13e‐02 (−1.06e‐01, −3.68e‐02)	1.15E‐04
	21‐hydroxypregnenolone disulfate	0.92	−5.96e‐02 (−9.15e‐02, −2.78e‐02)	4.39E‐04
	Pregnenediol sulfate (C21H34O5S)	0.92	−5e‐02 (−9.11e‐02, −8.84e‐03)	1.96E‐02
	Dehydroepiandrosterone sulfate (DHEA‐S)	0.91	−6.2e‐02 (−1.05e‐01, −1.93e‐02)	5.62E‐03
	Pregnenetriol sulfate	0.91	−5.59e‐02 (−1.05e‐01, −6.53e‐03)	2.93E‐02
	Pregnenolone sulfate	0.89	−7.68e‐02 (−1.23e‐01, −3.06e‐02)	1.64E‐03
	Pregnenediol disulfate (C21H34O8S2)	0.87	−9.61e‐02 (−1.29e‐01, −6.32e‐02)	1.78E‐07
	Androstenediol (3beta,17beta) disulfate (1)	0.86	−7.91e‐02 (−1.2e‐01, −3.76e‐02)	3.44E‐04
	Androstenediol (3beta,17beta) monosulfate (1)	0.85	−6.97e‐02 (−1.2e‐01, −1.9e‐02)	8.58E‐03
	Epiandrosterone sulfate	0.84	−6.84e‐02 (−1.35e‐01, −1.63e‐03)	4.80E‐02
	5alpha‐androstan‐3beta,17beta‐diol disulfate	0.82	−7.98e‐02 (−1.48e‐01, −1.11e‐02)	2.55E‐02
	Androsterone glucuronide	0.82	−5.62e‐02 (−1.04e‐01, −8.64e‐03)	2.31E‐02

MM, module‐membership.

^a^ MM for each metabolite is defined as the correlation of individual metabolites with the related eigen‐metabolite. Metabolites significantly changed during the study are displayed. The complete list of metabolites in magenta and green modules is in Table S2.

^b^ The *p*‐value is derived from using a linear mixed effect model with the metabolite level as the dependent and the duration from the baseline visit (measured in years) as the independent variable, adjusted for age at baseline, sex, and prior history of DMT.

### Association of metabolome alteration with disability outcome measures and quality of life

**Figure 3:**
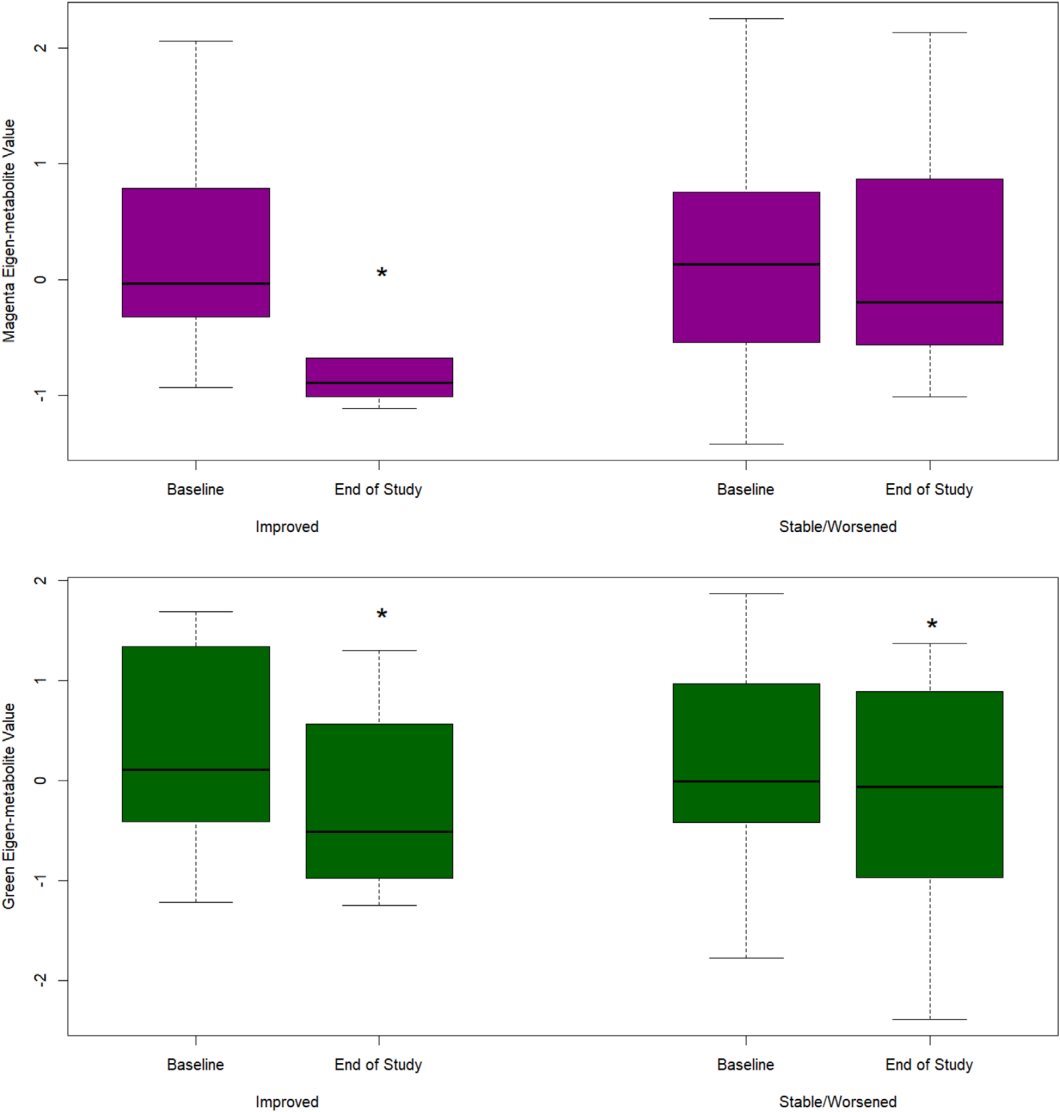
Green and Magenta eigen‐metabolite changes in improved and stable/worsened patients, the significance of the changes was determined using the Wilcoxon exact rank test, with significance level indicated by an asterisk '*' for a p‐value ≤ 0.05.

**Figure 4:**
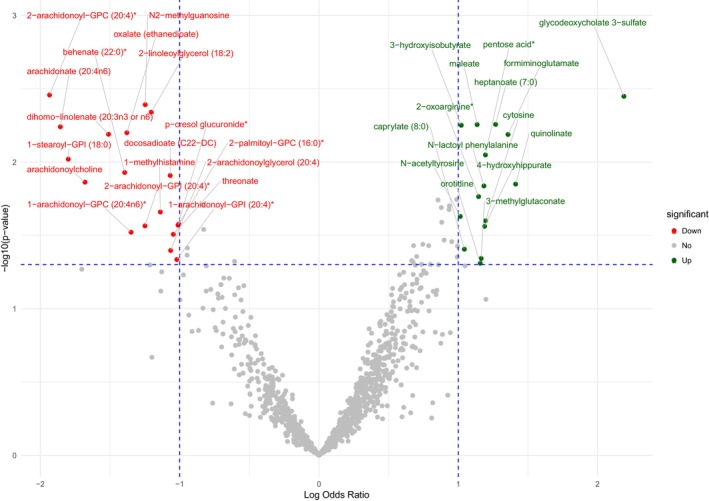
Volcano plot of metabolites whose change was associated with improvement in Overall Disability Response Score (ODRS).

To investigate the relationship between alterations in the circulating metabolome and patients' disease status, individuals were classified into improved and unimproved (containing both stable and deteriorating patients) groups. Nine patients were classified as improved based on the ODRS method. The result of the logistic regression model showed that only a change in the magenta eigen‐metabolite was significantly associated with the improvement of patients (OR 3.09e‐01, 95% CI: 6.83e‐02, 9.09e‐01, *p*‐value = 3.15E‐02) (Table S3). The same result was obtained when improvement was defined using the EDSS‐Plus, with eight patients being classified as improved (OR 6.11e‐02, 95% CI: 2.2e‐03, 4.46e‐01, *p*‐value = 2.68e‐02). The green eigen‐metabolite value was correlated with age (Coefficient: −0.58, *p*‐value <0.001), and its reduction was seen in both improved and unimproved patients, regardless of the disease status during the study. However, the reduction of the magenta eigen‐metabolite was exclusively observed in improved patients (Fig. 3, *p*‐value = 0.012). Using similar analyses assessing the association between individual metabolites and improvement, we observed that reductions in several LPL metabolites were strongly associated with improvement independent of age, sex, DMT history, and years of follow‐up (Table S4, Fig. 4).

**Figure 5:**
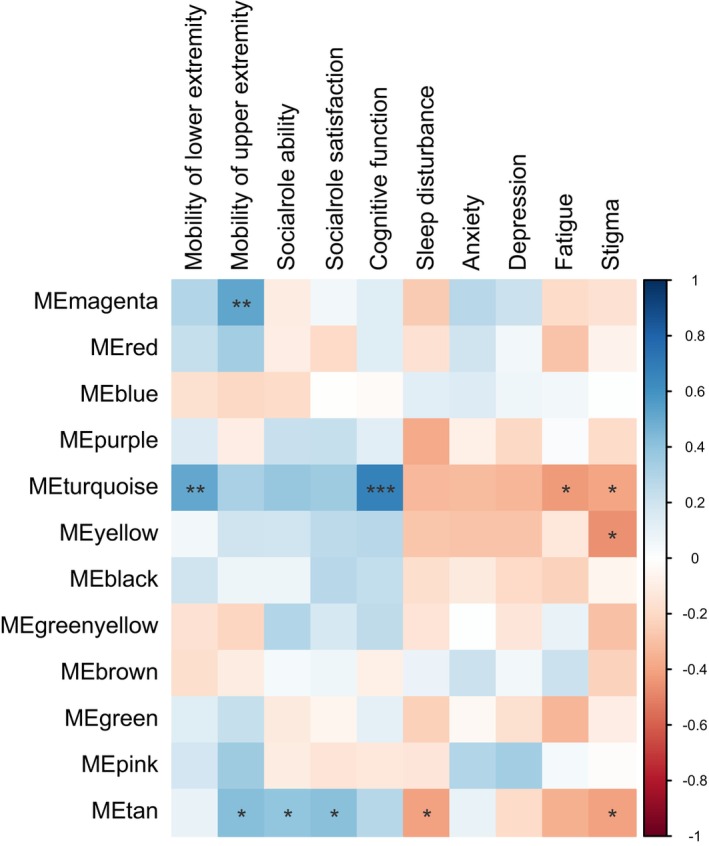
Heatmap of partial Spearman's correlation between changes in module eigen‐metabolites and changes in neuro‐QOL items, adjusted for patients' age at baseline, years of follow‐up, sex, and DMT history, with significance levels indicated by asterisks (*** for *p*‐value ≤0.001, ** for *p*‐value ≤0.01, * for *p*‐value ≤0.05).

Analysis of neuro‐QOL results aiming to investigate the relationship between quality of life and metabolic pathways showed that changes in the tan module (containing phosphatidylinositols and phosphatidylcholines) and turquoise module (containing long‐chain saturated and unsaturated fatty acids) eigen‐metabolites were correlated with change in patients' reported quality of life (Figure 5).

Analyses using logistic regression to identify associations with quality of life improvement demonstrated an increase in the tan module eigen‐metabolite was associated with the improvement of anxiety (OR 7.54e+00, 95% CI: 1.14e+00, 1.06e+02, *p*‐value = 3.44e‐02) and fatigue (OR 5.23e+00, 95% CI: 1.19e+00, 4.14e+01, *p*‐value = 2.62e‐02) in patients. Turquoise module (OR 2.2e+00, 95% CI: 1.04e+00, 6.26e+00, *p*‐value = 3.9e‐02), green module (OR 7.96e+00, 95% CI: 1.14e+00, 1.07e+02, *p*‐value = 3.48e‐02), and black module (mainly contained purine, pyrimidine, and amino acids metabolisms) (OR 3.14e+00, 95% CI: 1.12e+00, 1.24e+01, *p*‐value = 2.78e‐02) eigen‐metabolite were other factors associated with fatigue improvement.

## Discussion

This longitudinal observational study demonstrated significant alterations in the metabolome following ocrelizumab treatment in patients with RRMS using analyses based on either individual metabolites or metabolic pathways. The results of both the WGCNA pathway analysis and the individual metabolite analysis revealed significant changes in steroid metabolites and metabolites involved in LPL and monohydroxy fatty acid metabolism. Changes in lipid metabolism were significantly associated with changes in the quality of life and disability status of patients. In particular, the concentration of LPL metabolites was found to be a potential factor associated with improvement in patients' disease status.

Our primary analysis demonstrated that ocrelizumab treatment reduced the levels of multiple LPLs and monohydroxy fatty acids. Altered levels of various metabolites in lipid metabolism have been observed in PwMS.^24^, ^25^, ^26^ Notably, distinct changes in lipid metabolites were observed with dimethyl fumarate (DMF) compared to ocrelizumab treatment in patients with RRMS. Specifically, reduced circulating levels of fatty acid metabolites following DMF treatment were correlated with immune alterations. Additionally, increased levels of phospholipids, plasmalogens, and LPLs were observed, but these changes did not show any significant association with immune alterations.^7^ LPLs, especially lysophosphatidylcholines (LPC) have pro‐inflammatory, and cytotoxic characteristics by several mechanisms^27^, ^28^; They increase the production of inflammatory mediators,^29^ suppress the function of naturally occurring regulatory T cells (nTregs),^30^, ^31^ enhance the production of reactive oxygen species (ROS),^32^ and induce the chemotaxis in T cells and natural killer cells.^33^, ^34^ The production of chemotactic factors and increased generation of ROS was found to be associated with saturated LPC16:0 and LPC18:0^32^, ^35^; Notably, the reduction in LPC16:0 was associated with patients' improvement in our study. Given the demonstrated pro‐inflammatory properties of LPLs and their role in exacerbating the inflammatory cascade, decreased inflammation and stability in patients of our study could be partially attributed to the reduction of LPLs.

Increased activation of phospholipase A2 (PLA2) during inflammation leads to LPC and arachidonic acid (AA) production from phospholipids of the myelin sheath.^36^, ^37^, ^38^ LPCs induce oligodendrocyte demyelination leading to the exacerbation of demyelination.^39^, ^40^ Therefore, it is logical to see the reduction of LPCs together with the reduction of inflammation and demyelination. LPCs can induce proinflammatory activation of monocytes by initiating two signaling pathways including cPLA_2_, leading to increased release of AA.^41^, ^42^ Given that WGCNA analysis clusters metabolites that are related functionally, AA being clustered with other LPLs in the magenta module could be due to this important pathway. AA which is a polyunsaturated fatty acid that exists abundantly in the CNS and its pathway and derivative lipid mediators are shown to be associated with MS severity by their inflammatory and demyelinating properties.^43^, ^44^, ^45^, ^46^ The reduction of arachidonic acid together with several arachidonoyl LPCs highlights the important role of these metabolites in potentially exacerbating inflammation and progression in MS.

The treatment also impacted other metabolic pathways that were previously shown to be altered in MS. We saw increased levels of benzoate metabolites following treatment, and this increase was associated with patients' improvement. Reduced benzoate metabolites in PwMS compared to healthy people were observed in previous studies.^5^, ^18^ Although the exact role of benzoate and its metabolites is not clear, the neuroprotective property of sodium benzoate was shown in other neurological disorders including Parkinson's disease, Alzheimer's disease, and traumatic brain injury.^47^, ^48^, ^49^, ^50^ Moreover, higher sodium benzoate levels have recently been shown to be associated with the enrichment of proteins involved in lipid metabolism and immune responses in Alzheimer's disease.^51^

We also saw a notable reduction in androgenic and pregnenolone steroids. Many of the metabolites belonging to this group are neurosteroids which are known to possess immunomodulatory and neuroprotective properties.^52^ Impaired neurosteroid synthesis and its possible role in the neuroinflammatory process of MS have been demonstrated in studies.^52^, ^53^, ^54^ Since we hypothesized that the metabolome potentially normalizes following ocrelizumab, in this case, ocrelizumab was not effective in correcting the impaired steroidogenesis observed in MS. In our primary analysis, we observed a negative correlation between steroid levels and age. Given that most of our participants were females in their 40s, in their perimenopause stage, and our study had a long follow‐up duration, another likely explanation for the observed reduction in steroid levels could be attributed to the natural aging process, particularly as individuals approach menopausal age.^55^, ^56^

Two modules within our study, containing phospholipids and fatty acids, were correlated with improvement in patients' quality of life measures. This correlation could be mediated through various metabolites in these modules, known for their established anti‐inflammatory effects. For instance, docosahexaenoic acid (DHA), eicosapentaenoic acid (EPA), and docosapentaenoic acid (DPA) and their metabolites in these modules are recognized for anti‐inflammatory and neuroprotective properties,^57^, ^58^, ^59^, ^60^ which could be associated with improved quality of life. However, since these modules contain several kinds of phospholipids and fatty acids, making a precise determination of how their increase was associated with quality of life is challenging. The apparent dissociation between the metabolites associated with quality of life and those associated with disability measures could arise from two possible sources. The first is that there is a distinct biological basis for these relationships with different metabolites being linked to domains measured in quality of life like cognition and mood. Alternatively, given our limited sample size, this could be a function of inadequate power to detect a relationship of the tan and turquoise modules with disability outcomes or to detect a relationship between the magenta module and quality of life outcomes.

Although we conducted a longitudinal assessment of the effect ocrelizumab had on the circulating metabolome and addressed the limitation of cross‐sectional analyses, there are a few noteworthy limitations to our study. One significant limitation of our study is the absence of a control group, specifically the lack of a control group comprising PwMS using DMTs other than ocrelizumab, and/or healthy individuals. The inclusion of such a control group could have helped detect age, and treatment‐related alterations in the metabolome, as well as to differentiate metabolomic changes attributed specifically to the impact of peripheral B cell depletion versus a non‐specific decrease in MS disease activity. Moreover, at the time of starting our study, ocrelizumab was the only B‐cell depleting therapy approved for multiple sclerosis, and hence we could not compare metabolomic changes across different B‐cell depleting agents. Based on similar mechanisms of action of B‐cell depleting therapies, we would not expect significant differences in the overall impact on circulating metabolites between these agents. However, future research should explore the metabolomic effects of other B‐cell depleting therapies to validate and expand upon our findings.

Additionally, since we only included patients with RRMS and did not investigate the progressive forms of MS, we are unable to generalize our results on all MS types. Another limitation is that although we included all identified metabolites that were robustly detected in our analyses, we were unable to assess the association of short‐chain fatty acids (SCFAs) with disease status. This limitation arises because the global untargeted metabolomics method we employed is not optimized for detecting SCFAs, which requires a targeted methodology for accurate identification and quantification. Moreover, the relatively small sample size limited our ability to conduct sensitivity analyses. As a result, we were unable to separately evaluate the effect of the history of each type of DMT prior to ocrelizumab initiation on metabolome changes. A larger sample size would also enable us to perform longitudinal mediation analysis, providing valuable insights into the underlying mechanisms of the effect of ocrelizumab on the metabolome and outcomes, as well as a sensitive analysis by classifying patients as deteriorating or otherwise (stable plus improving). To address these concerns properly, future longitudinal studies with control groups and larger sample sizes are necessary.

## Conclusion

In this longitudinal observational study, we demonstrated significant circulating metabolome alterations in RRMS patients as a result of ocrelizumab treatment using a global untargeted metabolomics approach. In particular, metabolites involved in the lysophospholipid pathway were reduced significantly, which was associated with improvement in patients' disability measures. The findings of our study serve as a rationale for future follow‐up studies to develop biomarkers for prognosis and treatment response to B cell‐depleting therapies.

## Conflict of Interest

Dr. Bhargava has received honoraria from EMD‐Serono and Genentech, and funding support from EMD‐Serono, Genentech, GSK, and Amylyx Pharmaceuticals outside the submitted work. Dr. Nourbakhsh has received grants from the US Department of Defense, Genentech, the National Multiple Sclerosis Society, and personal fees for Alkermes, honoraria from TG Therapeutics outside the submitted work. Dr. Siavoshi, Dr. Ladakis, and Ms. Muller report no disclosures.

## Author Contributions

PB and BN contributed to the conception and design of the study. PB, BN, FS, DCL, and AM contributed to the acquisition and analysis of the data. PB, BN, FS, DCL, and AM contributed to drafting and revising the manuscript.

## Supporting information


Data S1.

Table S1.

## Data Availability

The data that support the findings of this study are available from the corresponding author upon reasonable request.
