# Supplementary material for: Ocrelizumab alters the circulating metabolome in people with relapsing–remitting multiple sclerosis
Source: Ann Clin Transl Neurol. 2024 Aug 26;11(9):2485–98. doi: 10.1002/acn3.52167 (PMC11537130; doi:10.1002/acn3.52167)
Supplement: Supplementary file 1 — Data S1. [file ACN3-11-2485-s001.docx]

**Supplementary Methods**

Covariate Selection Process

To balance the inclusion of necessary covariates we conducted a covariate selection process. Initially, we fit univariate models for each covariate (including baseline EDSS, sex, prior history of DMT, baseline BMI, and baseline age) with each module eigen-metabolite to assess their individual impacts. Considering that over 90% of participants in our study were Caucasian, we did not adjust our models for race and ethnicity.

For the linear mixed-effects (LME) model examining the association between ocrelizumab treatment and metabolome change, we proceeded as follows:

- We fit univariate models for each aforementioned covariate and module eigen-metabolite.
- After fitting the models, we assessed the effects of the covariates across all models. Covariates with negligible effects across most models were considered for exclusion.

Based on previous studies, the influence of the covariates, and considering our sample size, we selected sex, prior history of DMT, and age at baseline for our module eigen-metabolite models, and did not include baseline EDSS and BMI as covariates since they did not significantly alter the results. To ensure interpretability and consistency, we used the same set of covariates across all metabolite models. This approach simplified comparisons between the results of the network correlation analysis (models using module eigen-metabolite) and individual metabolite analysis, ensuring that possible differences were not due to varying model specifications.

Although including baseline BMI and baseline EDSS as covariates did not significantly alter the results, because of their potential importance we have provided the results of the LME models with these covariates included.

**Change of Eigen-metabolite Values in Linear Mixed-Effect Models over the time of study:**

| Module | Estimate^1^ | P-value^1^ | Estimate^2^ | P-value^2^ |
| --- | --- | --- | --- | --- |
| Magenta | -0.126 | **0.016** | -0.125 | **0.018** |
| Red | -0.069 | 0.054 | -0.067 | 0.063 |
| Blue | 0.039 | 0.368 | 0.040 | 0.363 |
| Purple | -0.100 | 0.082 | -0.103 | 0.074 |
| Turquoise | -0.033 | 0.555 | -0.035 | 0.531 |
| Yellow | -0.079 | 0.064 | -0.084 | 0.051 |
| Black | -0.019 | 0.721 | -0.023 | 0.662 |
| Greenyellow | -0.066 | 0.199 | -0.069 | 0.179 |
| Brown | 0.017 | 0.741 | 0.007 | 0.885 |
| Green | -0.106 | **<0.001** | -0.107 | **<0.001** |
| Pink | -0.062 | 0.146 | -0.060 | 0.154 |
| Tan | -0.057 | 0.083 | -0.057 | 0.081 |

1. Estimate and P-value derived from Linear Mixed-Effect Models adjusting for age at baseline, sex, years of follow-up, and DMT history

2. Estimate and P-value derived from Linear Mixed-Effect Models adjusting for age at baseline, sex, years of follow-up, DMT history, baseline BMI, and baseline EDSS score

In the logistic regression analysis examining the association between metabolite change following ocrelizumab treatment and clinical and quality of life improvement, we applied a similar approach, with the only difference being the inclusion of years of follow-up among the potential covariates. Based on previous studies, and the influence of the covariates, we selected sex, prior history of DMT, age at baseline, and years of follow-up for our module eigen-metabolite models. To ensure interpretability and consistency, we used the same set of covariates across all metabolite models. In the following section, we have provided the results of the logistic regression models, both with and without baseline BMI and baseline EDSS as covariates for comparison. We encountered convergence issues while fitting some of the logistic regression models that used NeuroQoL items such as sleep disturbance, cognitive performance, social satisfaction, and upper and lower extremity mobility as response variables, due to the insufficient sample size in the improved group for each of these items. As a result, we have not provided the results for those items.

**Logistic regression results to assess the association between module eigenmetabolite change and odds of improvement based on ODRS:**

| Module | Odds Ratio for 1SD (95% CI)^1^ | P-value^1^ | Odds Ratio for 1SD (95% CI)^2^ | P-value^2^ |
| --- | --- | --- | --- | --- |
| Magenta | 3.09e-01 (6.83e-02, 9.09e-01) | **3.15E-02** | 3.27e-01 (7.62e-02, 9.42e-01) | **3.74E-02** |
| Purple | 1.74e+00 (9.13e-01, 4.54e+00) | 9.76E-02 | 2.28e+00 (1.07e+00, 6.77e+00) | **3.13E-02** |
| Blue | 1.98e+00 (7.9e-01, 6.43e+00) | 1.51E-01 | 1.99e+00 (7.4e-01, 6.75e+00) | 1.79E-01 |
| Greenyellow | 1.36e+00 (6.84e-01, 3.01e+00) | 3.84E-01 | 1.34e+00 (6.82e-01, 2.9e+00) | 3.98E-01 |
| Tan | 5.96e-01 (1.41e-01, 2.19e+00) | 4.39E-01 | 6.17e-01 (1.52e-01, 2.2e+00) | 4.59E-01 |
| Black | 1.32e+00 (5.39e-01, 3.68e+00) | 5.46E-01 | 1.27e+00 (5.32e-01, 3.48e+00) | 5.90E-01 |
| Pink | 7.71e-01 (2.93e-01, 1.94e+00) | 5.76E-01 | 7.66e-01 (2.97e-01, 1.9e+00) | 5.60E-01 |
| Brown | 1.22e+00 (5.91e-01, 2.64e+00) | 5.85E-01 | 1.2e+00 (5.72e-01, 2.61e+00) | 6.31E-01 |
| Red | 8.22e-01 (2.77e-01, 2.42e+00) | 7.09E-01 | 7.78e-01 (2.67e-01, 2.34e+00) | 6.38E-01 |
| Turquoise | 8.92e-01 (4.54e-01, 1.76e+00) | 7.35E-01 | 9.19e-01 (4.64e-01, 1.86e+00) | 8.05E-01 |
| Yellow | 1.09e+00 (3.76e-01, 3.2e+00) | 8.70E-01 | 1.06e+00 (3.63e-01, 3.15e+00) | 9.09E-01 |
| Green | 1e+00 (1.64e-01, 6.35e+00) | 9.96E-01 | 1.07e+00 (1.76e-01, 7.3e+00) | 9.39E-01 |

1. Odds ratio and P-value derived from logistic regression models on one SD change in module eigenmetabolite value adjusting for age at baseline, sex, years of follow-up, and DMT history

2. P-value derived from logistic regression models on one SD change in module eigenmetabolite value adjusting for age at baseline, sex, years of follow-up, DMT history, baseline BMI, and baseline EDSS score

**Logistic regression results to assess the association between module eigenmetabolite change and odds of fatigue improvement based on the NeuroQoL questionnaire:**

| Module | Odds Ratio for 1SD (95% CI)^1^ | P-value^1^ | Odds Ratio for 1SD (95% CI)^2^ | P-value^2^ |
| --- | --- | --- | --- | --- |
| Tan | 5.23e+00 (1.19e+00, 4.14e+01) | **2.62E-02** | 4.6e+00 (9.83e-01, 4.59e+01) | 5.28E-02 |
| Black | 3.14e+00 (1.12e+00, 1.24e+01) | **2.78E-02** | 3e+00 (1.03e+00, 1.23e+01) | **4.33E-02** |
| Green | 7.96e+00 (1.14e+00, 1.07e+02) | **3.48E-02** | 3.9e+00 (6.37e-01, 4.79e+01) | 1.48E-01 |
| Turquoise | 2.2e+00 (1.04e+00, 6.26e+00) | **3.90E-02** | 2.11e+00 (9.02e-01, 8.17e+00) | 8.95E-02 |
| Yellow | 1.5e+00 (5.66e-01, 4.28e+00) | 4.15E-01 | 1.32e+00 (4.65e-01, 4.08e+00) | 5.94E-01 |
| Pink | 6.84e-01 (2.37e-01, 1.78e+00) | 4.37E-01 | 7.76e-01 (2.71e-01, 2.06e+00) | 6.08E-01 |
| Purple | 1.22e+00 (6.76e-01, 2.39e+00) | 5.22E-01 | 8.61e-01 (3.64e-01, 1.95e+00) | 7.21E-01 |
| Magenta | 1.33e+00 (5.06e-01, 3.93e+00) | 5.62E-01 | 1.36e+00 (5.19e-01, 3.91e+00) | 5.27E-01 |
| Brown | 8.78e-01 (4.03e-01, 1.91e+00) | 7.35E-01 | 1.06e+00 (4.51e-01, 2.64e+00) | 8.94E-01 |
| Blue | 8.62e-01 (3.37e-01, 2.14e+00) | 7.45E-01 | 1.22e+00 (4.29e-01, 3.73e+00) | 7.07E-01 |
| Red | 8.75e-01 (2.95e-01, 2.64e+00) | 8.02E-01 | 1e+00 (3.31e-01, 3.1e+00) | 9.97E-01 |
| Greenyellow | 1.07e+00 (5.48e-01, 2.22e+00) | 8.34E-01 | 1.15e+00 (5.62e-01, 2.64e+00) | 7.12E-01 |

1. P-value derived from logistic regression models on one SD change in module eigenmetabolite value adjusting for age at baseline, sex, years of follow-up, and DMT history

2. P-value derived from logistic regression models on one SD change in module eigenmetabolite value adjusting for age at baseline, sex, years of follow-up, DMT history, baseline BMI, and baseline EDSS score

**Logistic regression results to assess the association between module eigenmetabolite change and odds of anxiety improvement based on the NeuroQoL questionnaire:**

| Module | Odds Ratio for 1SD (95% CI)^1^ | P-value^1^ | Odds Ratio for 1SD (95% CI)^2^ | P-value^2^ |
| --- | --- | --- | --- | --- |
| Tan | 7.54e+00 (1.14e+00, 1.06e+02) | **3.44E-02** | 9.88e+00 (8.52e-01, 1.01e+03) | 7.23E-02 |
| Turquoise | 2.17e+00 (8.84e-01, 8.11e+00) | 9.67E-02 | 2.21e+00 (8.39e-01, 1.08e+01) | 1.22E-01 |
| Pink | 4.12e-01 (8.87e-02, 1.24e+00) | 1.19E-01 | 4.47e-01 (9.95e-02, 1.29e+00) | 1.41E-01 |
| Blue | 4.57e-01 (1.29e-01, 1.28e+00) | 1.39E-01 | 5.22e-01 (1.44e-01, 1.51e+00) | 2.33E-01 |
| Red | 4.79e-01 (1.32e-01, 1.55e+00) | 2.13E-01 | 4.89e-01 (1.37e-01, 1.59e+00) | 2.24E-01 |
| Yellow | 1.49e+00 (5.35e-01, 4.43e+00) | 4.43E-01 | 1.34e+00 (4.63e-01, 4.22e+00) | 5.94E-01 |
| Greenyellow | 1.31e+00 (6.41e-01, 3e+00) | 4.67E-01 | 1.25e+00 (6.37e-01, 2.75e+00) | 5.19E-01 |
| Purple | 1.31e+00 (6.38e-01, 3.29e+00) | 4.79E-01 | 1.21e+00 (4.98e-01, 3.14e+00) | 6.68E-01 |
| Magenta | 7.57e-01 (2.3e-01, 2.16e+00) | 6.03E-01 | 7.61e-01 (2.36e-01, 2.15e+00) | 6.01E-01 |
| Black | 1.19e+00 (4.69e-01, 3.33e+00) | 7.12E-01 | 1.15e+00 (4.52e-01, 3.12e+00) | 7.65E-01 |
| Brown | 8.72e-01 (3.27e-01, 2.45e+00) | 7.75E-01 | 9.32e-01 (3.46e-01, 2.63e+00) | 8.82E-01 |
| Green | 1.24e+00 (1.94e-01, 9.57e+00) | 8.22E-01 | 8.35e-01 (1.38e-01, 6.65e+00) | 8.48E-01 |

1. P-value derived from logistic regression models on one SD change in module eigenmetabolite value adjusting for age at baseline, sex, years of follow-up, and DMT history

2. P-value derived from logistic regression models on one SD change in module eigenmetabolite value adjusting for age at baseline, sex, years of follow-up, DMT history, baseline BMI, and baseline EDSS score

**Logistic regression results to assess the association between module eigenmetabolite change and odds of depression improvement based on the NeuroQoL questionnaire:**

| Module | Odds Ratio for 1SD (95% CI)^1^ | P-value^1^ | Odds Ratio for 1SD (95% CI)^2^ | P-value^2^ |
| --- | --- | --- | --- | --- |
| Turquoise | 1.86e+00 (9.1e-01, 4.7e+00) | 9.15E-02 | 1.61e+00 (7.65e-01, 4.45e+00) | 2.21E-01 |
| Tan | 2.7e+00 (7.26e-01, 1.32e+01) | 1.43E-01 | 2.28e+00 (5.59e-01, 1.2e+01) | 2.51E-01 |
| Yellow | 2e+00 (7.61e-01, 6.16e+00) | 1.63E-01 | 1.58e+00 (5.79e-01, 4.96e+00) | 3.74E-01 |
| Pink | 5.52e-01 (2.01e-01, 1.38e+00) | 2.04E-01 | 6.07e-01 (2.25e-01, 1.55e+00) | 2.92E-01 |
| Red | 5.82e-01 (1.9e-01, 1.71e+00) | 3.11E-01 | 5.58e-01 (1.72e-01, 1.72e+00) | 2.95E-01 |
| Magenta | 6.41e-01 (2.23e-01, 1.59e+00) | 3.40E-01 | 6.67e-01 (2.24e-01, 1.68e+00) | 3.90E-01 |
| Blue | 6.79e-01 (2.66e-01, 1.6e+00) | 3.76E-01 | 7.83e-01 (2.87e-01, 2.06e+00) | 6.15E-01 |
| Greenyellow | 1.31e+00 (6.85e-01, 2.73e+00) | 4.20E-01 | 1.37e+00 (7.11e-01, 3e+00) | 3.56E-01 |
| Black | 1.3e+00 (5.5e-01, 3.46e+00) | 5.54E-01 | 1.18e+00 (4.9e-01, 3.04e+00) | 7.05E-01 |
| Purple | 1.18e+00 (6.38e-01, 2.41e+00) | 6.05E-01 | 1.05e+00 (4.46e-01, 2.39e+00) | 9.04E-01 |
| Green | 1.4e+00 (2.45e-01, 9.46e+00) | 7.07E-01 | 9.14e-01 (1.51e-01, 5.65e+00) | 9.18E-01 |
| Brown | 1.04e+00 (4.7e-01, 2.44e+00) | 9.20E-01 | 1.26e+00 (5.37e-01, 3.54e+00) | 5.93E-01 |

1. P-value derived from logistic regression models on one SD change in module eigenmetabolite value adjusting for age at baseline, sex, years of follow-up, and DMT history

2. P-value derived from logistic regression models on one SD change in module eigenmetabolite value adjusting for age at baseline, sex, years of follow-up, DMT history, baseline BMI, and baseline EDSS score

**Logistic regression results to assess the association between module eigenmetabolite change and odds of stigma improvement based on the NeuroQoL questionnaire:**

| Module | Odds Ratio for 1SD (95% CI)^1^ | P-value^1^ | Odds Ratio for 1SD (95% CI)^2^ | P-value^2^ |
| --- | --- | --- | --- | --- |
| Black | 4.96e-01 (1.66e-01, 1.22e+00) | 1.28E-01 | 5.29e-01 (1.95e-01, 1.24e+00) | 1.44E-01 |
| Yellow | 1.72e+00 (6.37e-01, 5.27e+00) | 2.87E-01 | 1.78e+00 (6.27e-01, 5.76e+00) | 2.83E-01 |
| Turquoise | 1.49e+00 (7.27e-01, 3.58e+00) | 2.87E-01 | 1.54e+00 (7.34e-01, 3.75e+00) | 2.66E-01 |
| Tan | 2.08e+00 (5.31e-01, 1.08e+01) | 2.99E-01 | 2.22e+00 (5.61e-01, 1.67e+01) | 2.66E-01 |
| Purple | 7.3e-01 (3.74e-01, 1.38e+00) | 3.22E-01 | 7.38e-01 (3.33e-01, 1.5e+00) | 3.96E-01 |
| Blue | 6.61e-01 (2.51e-01, 1.64e+00) | 3.70E-01 | 5.84e-01 (1.99e-01, 1.55e+00) | 2.79E-01 |
| Green | 4.55e-01 (6.33e-02, 2.73e+00) | 3.86E-01 | 4.72e-01 (6.83e-02, 2.91e+00) | 4.07E-01 |
| Greenyellow | 1.36e+00 (6.78e-01, 3.05e+00) | 3.90E-01 | 1.36e+00 (6.84e-01, 3.03e+00) | 3.88E-01 |
| Red | 8.02e-01 (2.62e-01, 2.8e+00) | 7.01E-01 | 7.5e-01 (2.38e-01, 2.6e+00) | 6.23E-01 |
| Pink | 8.34e-01 (3.09e-01, 2.19e+00) | 7.06E-01 | 8.23e-01 (3.11e-01, 2.13e+00) | 6.82E-01 |
| Magenta | 8.37e-01 (3e-01, 2.18e+00) | 7.10E-01 | 8.42e-01 (3.1e-01, 2.14e+00) | 7.13E-01 |
| Brown | 1.02e+00 (4.37e-01, 2.53e+00) | 9.62E-01 | 1.01e+00 (4.38e-01, 2.54e+00) | 9.79E-01 |

1. P-value derived from logistic regression models on one SD change in module eigenmetabolite value adjusting for age at baseline, sex, years of follow-up, and DMT history

2. P-value derived from logistic regression models on one SD change in module eigenmetabolite value adjusting for age at baseline, sex, years of follow-up, DMT history, baseline BMI, and baseline EDSS score

**Logistic regression results to assess the association between module eigenmetabolite change and odds of social role ability improvement based on the NeuroQoL questionnaire:**

| Module | Odds Ratio for 1SD (95% CI)^1^ | P-value^1^ | Odds Ratio for 1SD (95% CI)^2^ | P-value^2^ |
| --- | --- | --- | --- | --- |
| Turquoise | 1.79e+00 (9.32e-01, 3.99e+00) | 8.16E-02 | 1.93e+00 (9.6e-01, 4.7e+00) | 6.59E-02 |
| Blue | 4.7e-01 (1.69e-01, 1.1e+00) | 8.44E-02 | 4.31e-01 (1.54e-01, 1.07e+00) | 6.97E-02 |
| Tan | 2.54e+00 (7.63e-01, 9.86e+00) | 1.31E-01 | 2.46e+00 (7.59e-01, 9.34e+00) | 1.36E-01 |
| Greenyellow | 1.66e+00 (8.64e-01, 3.68e+00) | 1.32E-01 | 1.62e+00 (8.59e-01, 3.55e+00) | 1.40E-01 |
| Yellow | 2.05e+00 (7.99e-01, 6.31e+00) | 1.39E-01 | 2.23e+00 (8.27e-01, 7.35e+00) | 1.16E-01 |
| Purple | 1.22e+00 (7.08e-01, 2.26e+00) | 4.72E-01 | 1.32e+00 (6.97e-01, 2.75e+00) | 3.97E-01 |
| Red | 7.02e-01 (2.12e-01, 1.99e+00) | 5.03E-01 | 6.97e-01 (2.08e-01, 2e+00) | 5.04E-01 |
| Magenta | 7.95e-01 (3.1e-01, 1.93e+00) | 6.09E-01 | 8.03e-01 (3.2e-01, 1.92e+00) | 6.18E-01 |
| Pink | 8.03e-01 (2.92e-01, 2.02e+00) | 6.41E-01 | 8.12e-01 (3e-01, 2e+00) | 6.52E-01 |
| Brown | 9.36e-01 (4.62e-01, 1.89e+00) | 8.50E-01 | 9.38e-01 (4.68e-01, 1.88e+00) | 8.55E-01 |
| Green | 1.11e+00 (2.32e-01, 5.51e+00) | 8.94E-01 | 1.12e+00 (2.27e-01, 5.81e+00) | 8.85E-01 |
| Black | 9.65e-01 (4.37e-01, 2.16e+00) | 9.28E-01 | 9.76e-01 (4.47e-01, 2.17e+00) | 9.50E-01 |

1. P-value derived from logistic regression models on one SD change in module eigenmetabolite value adjusting for age at baseline, sex, years of follow-up, and DMT history

2. P-value derived from logistic regression models on one SD change in module eigenmetabolite value adjusting for age at baseline, sex, years of follow-up, DMT history, baseline BMI, and baseline EDSS score
