# Supplementary material for: Ocrelizumab alters the circulating metabolome in people with relapsing–remitting multiple sclerosis
Source: Ann Clin Transl Neurol. 2024 Aug 26;11(9):2485–98. doi: 10.1002/acn3.52167 (PMC11537130; doi:10.1002/acn3.52167)
Supplement: Supplementary file 2 — Table S1. [file ACN3-11-2485-s002.docx]

**Supplementary Table 1.** Metabolites changed significantly following treatment^1^

| Metabolic Pathway | Metabolite | Estimate (95% CI) | P-value^2^ | FDR P-value |
| --- | --- | --- | --- | --- |
| Androgenic Steroids | androstenediol (3beta,17beta) disulfate (2) | -7.13e-02 (-1.06e-01, -3.68e-02) | 1.15E-04 | 1.76E-02 |
|  | androstenediol (3beta,17beta) disulfate (1) | -7.91e-02 (-1.2e-01, -3.76e-02) | 3.44E-04 | 2.83E-02 |
|  | 16alpha-hydroxy DHEA 3-sulfate | -9.59e-02 (-1.52e-01, -3.97e-02) | 1.25E-03 | 7.30E-02 |
|  | androstenediol (3beta,17beta) monosulfate (2) | -8.56e-02 (-1.39e-01, -3.24e-02) | 2.25E-03 | 9.26E-02 |
|  | dehydroepiandrosterone sulfate (DHEA-S) | -6.2e-02 (-1.05e-01, -1.93e-02) | 5.62E-03 | 1.25E-01 |
|  | androstenediol (3beta,17beta) monosulfate (1) | -6.97e-02 (-1.2e-01, -1.9e-02) | 8.58E-03 | 1.58E-01 |
|  | androsterone glucuronide | -5.62e-02 (-1.04e-01, -8.64e-03) | 2.31E-02 | 2.52E-01 |
|  | 5alpha-androstan-3beta,17beta-diol disulfate | -7.98e-02 (-1.48e-01, -1.11e-02) | 2.55E-02 | 2.67E-01 |
|  | androsterone sulfate | -7.29e-02 (-1.41e-01, -4.36e-03) | 4.03E-02 | 3.27E-01 |
|  | androstenediol (3alpha, 17alpha) monosulfate (2) | -6.42e-02 (-1.25e-01, -3.04e-03) | 4.29E-02 | 3.30E-01 |
|  | epiandrosterone sulfate | -6.84e-02 (-1.35e-01, -1.63e-03) | 4.80E-02 | 3.54E-01 |
| Chemical | perfluorooctanesulfonate (PFOS) | -1.45e-01 (-1.99e-01, -9.08e-02) | 1.17E-06 | 6.23E-04 |
|  | perfluorooctanoate (PFOA) | -1.11e-01 (-1.68e-01, -5.49e-02) | 2.20E-04 | 2.16E-02 |
|  | perfluorohexanesulfonate (PFHxS) | -1.15e-01 (-1.79e-01, -5.08e-02) | 7.29E-04 | 4.91E-02 |
|  | 3,5-dichloro-2,6-dihydroxybenzoic acid | -8.96e-02 (-1.4e-01, -3.96e-02) | 7.36E-04 | 4.91E-02 |
|  | 3-bromo-5-chloro-2,6-dihydroxybenzoic acid | -8.87e-02 (-1.59e-01, -1.87e-02) | 1.51E-02 | 2.22E-01 |
|  | (2-butoxyethoxy) acetic acid | -1.08e-01 (-1.94e-01, -2.14e-02) | 1.67E-02 | 2.35E-01 |
|  | 4-hydroxychlorothalonil | -7.16e-02 (-1.3e-01, -1.34e-02) | 1.82E-02 | 2.48E-01 |
|  | 2-naphthol sulfate | 9.55e-02 (1.61e-02, 1.75e-01) | 2.08E-02 | 2.51E-01 |
| Fatty Acid, Monohydroxy | 2-hydroxylaurate | -5.91e-02 (-9.41e-02, -2.41e-02) | 1.40E-03 | 7.30E-02 |
|  | 2-hydroxyarachidate | -7.35e-02 (-1.22e-01, -2.5e-02) | 3.94E-03 | 1.15E-01 |
|  | 2-hydroxynervonate | -4.35e-02 (-7.92e-02, -7.83e-03) | 1.92E-02 | 2.49E-01 |
|  | 3-hydroxyhexanoate | -7.99e-02 (-1.46e-01, -1.39e-02) | 2.01E-02 | 2.49E-01 |
|  | 2-hydroxystearate | -3.06e-02 (-5.64e-02, -4.92e-03) | 2.21E-02 | 2.51E-01 |
|  | 2-hydroxyheptanoate | -4.14e-02 (-7.73e-02, -5.51e-03) | 2.65E-02 | 2.72E-01 |
|  | 2-hydroxybehenate | -4.2e-02 (-8.22e-02, -1.67e-03) | 4.45E-02 | 3.37E-01 |
| Lysophospholipid | 1-linoleoyl-GPA (18:2) | -9.76e-02 (-1.47e-01, -4.79e-02) | 2.39E-04 | 2.16E-02 |
|  | 2-docosahexaenoyl-GPC (22:6) | -7.86e-02 (-1.26e-01, -3.14e-02) | 1.63E-03 | 7.30E-02 |
|  | 1-docosahexaenoyl-GPC (22:6) | -4.29e-02 (-7.5e-02, -1.08e-02) | 1.06E-02 | 1.74E-01 |
|  | 2-linoleoyl-GPC (18:2) | -3.85e-02 (-6.88e-02, -8.12e-03) | 1.51E-02 | 2.22E-01 |
|  | 2-palmitoyl-GPC (16:0) | -4.2e-02 (-7.61e-02, -7.82e-03) | 1.83E-02 | 2.48E-01 |
|  | 2-arachidonoyl-GPE (20:4) | -4.73e-02 (-8.84e-02, -6.18e-03) | 2.69E-02 | 2.73E-01 |
|  | 1-stearoyl-GPG (18:0) | -4.67e-02 (-8.96e-02, -3.88e-03) | 3.56E-02 | 2.99E-01 |
| Phosphatidylcholine (PC) | 1-palmitoyl-2-docosahexaenoyl-GPC (16:0/22:6) | -2.88e-02 (-4.84e-02, -9.22e-03) | 5.05E-03 | 1.25E-01 |
|  | 1-margaroyl-2-linoleoyl-GPC (17:0/18:2) | -2.78e-02 (-4.83e-02, -7.24e-03) | 9.68E-03 | 1.67E-01 |
|  | 1-stearoyl-2-(hydroxylinoleoyl)-GPC (18:0/18:2(OH)) | -3.7e-02 (-6.8e-02, -6.07e-03) | 2.15E-02 | 2.51E-01 |
|  | 1-oleoyl-2-docosahexaenoyl-GPC (18:1/22:6) | -2.69e-02 (-5.08e-02, -2.94e-03) | 3.06E-02 | 2.84E-01 |
|  | 1-palmitoyl-2-(hydroxylinoleoyl)-GPC (16:0/18:2(OH)) | -3.08e-02 (-5.99e-02, -1.69e-03) | 4.13E-02 | 3.27E-01 |
|  | 1,2-dipalmitoyl-GPC (16:0/16:0) | -1.32e-02 (-2.6e-02, -3.61e-04) | 4.72E-02 | 3.51E-01 |
|  | 1-pentadecanoyl-2-docosahexaenoyl-GPC (15:0/22:6) | -3.62e-02 (-7.17e-02, -6.37e-04) | 4.94E-02 | 3.59E-01 |
| Benzoate Metabolism | propyl 4-hydroxybenzoate sulfate | 5.93e-01 (3.46e-01, 8.39e-01) | 1.02E-05 | 3.64E-03 |
|  | 4-ethylphenylsulfate | 1.94e-01 (6.61e-02, 3.23e-01) | 3.94E-03 | 1.15E-01 |
|  | 3-methoxycatechol sulfate (2) | 1.19e-01 (3.96e-02, 1.98e-01) | 4.31E-03 | 1.15E-01 |
|  | 3-hydroxyhippurate | 1.61e-01 (4.63e-02, 2.76e-01) | 7.37E-03 | 1.41E-01 |
|  | guaiacol sulfate | 1.12e-01 (2.51e-02, 1.99e-01) | 1.35E-02 | 2.09E-01 |
|  | catechol sulfate | 7.23e-02 (8.47e-03, 1.36e-01) | 2.92E-02 | 2.84E-01 |
| Food Component/Plant | dihydrocaffeate sulfate (2) | 1.73e-01 (5.87e-02, 2.88e-01) | 3.99E-03 | 1.15E-01 |
|  | piperine | -1.37e-01 (-2.52e-01, -2.1e-02) | 2.32E-02 | 2.52E-01 |
|  | 3-ethylcatechol sulfate (1) | 1.62e-01 (1.76e-02, 3.07e-01) | 3.09E-02 | 2.84E-01 |
|  | 2,3-dihydroxyisovalerate | 1.69e-01 (1.75e-02, 3.2e-01) | 3.17E-02 | 2.87E-01 |
|  | phytanate | -9.48e-02 (-1.82e-01, -7.34e-03) | 3.67E-02 | 3.04E-01 |
|  | 4-acetylcatechol sulfate (1) | 1.1e-01 (6.53e-03, 2.14e-01) | 4.04E-02 | 3.27E-01 |
| Pregnenolone Steroids | pregnenediol disulfate (C21H34O8S2) | -9.61e-02 (-1.29e-01, -6.32e-02) | 1.78E-07 | 1.90E-04 |
|  | pregnenetriol disulfate | -7.65e-02 (-1.12e-01, -4.14e-02) | 5.29E-05 | 1.13E-02 |
|  | 21-hydroxypregnenolone disulfate | -5.96e-02 (-9.15e-02, -2.78e-02) | 4.39E-04 | 3.35E-02 |
|  | pregnenolone sulfate | -7.68e-02 (-1.23e-01, -3.06e-02) | 1.64E-03 | 7.30E-02 |
|  | pregnenediol sulfate (C21H34O5S) | -5e-02 (-9.11e-02, -8.84e-03) | 1.96E-02 | 2.49E-01 |
|  | pregnenetriol sulfate | -5.59e-02 (-1.05e-01, -6.53e-03) | 2.93E-02 | 2.84E-01 |
| Fibrinogen Cleavage Peptide | fibrinopeptide A (4-15) | -8.93e-02 (-1.43e-01, -3.61e-02) | 1.49E-03 | 7.30E-02 |
|  | fibrinopeptide A (8-16) | -8.19e-02 (-1.33e-01, -3.1e-02) | 2.25E-03 | 9.26E-02 |
|  | fibrinopeptide A (3-15) | -5.91e-02 (-9.99e-02, -1.84e-02) | 5.68E-03 | 1.25E-01 |
|  | fibrinopeptide A, phosphono-ser(3) | 5.34e-02 (8.91e-03, 9.79e-02) | 2.11E-02 | 2.51E-01 |
|  | fibrinopeptide A (7-16) | -4.1e-02 (-8.11e-02, -8.22e-04) | 4.89E-02 | 3.58E-01 |
| Partially Characterized Molecules | glutamine conjugate of C7H12O2 | -1.8e-01 (-3.01e-01, -6.02e-02) | 4.28E-03 | 1.15E-01 |
|  | glutamine conjugate of C6H10O2 (1) | -1.17e-01 (-1.97e-01, -3.58e-02) | 5.88E-03 | 1.25E-01 |
|  | bilirubin degradation product, C16H18N2O5 (3) | -6.23e-02 (-1.06e-01, -1.91e-02) | 5.98E-03 | 1.25E-01 |
|  | 2-amino-4-cyanobutanoate | 4.9e-02 (5.6e-03, 9.25e-02) | 2.98E-02 | 2.84E-01 |
|  | bilirubin degradation product, C16H18N2O5 (2) | -7.3e-02 (-1.38e-01, -7.74e-03) | 3.13E-02 | 2.85E-01 |
| Sphingomyelins | sphingomyelin (d18:2/18:1) | -4.79e-02 (-7.96e-02, -1.62e-02) | 4.05E-03 | 1.15E-01 |
|  | sphingomyelin (d18:1/22:2, d18:2/22:1, d16:1/24:2) | -3.32e-02 (-5.58e-02, -1.07e-02) | 5.01E-03 | 1.25E-01 |
|  | sphingomyelin (d18:2/24:2) | -3.11e-02 (-5.25e-02, -9.76e-03) | 5.47E-03 | 1.25E-01 |
|  | sphingomyelin (d18:2/23:1) | -3.69e-02 (-6.88e-02, -4.97e-03) | 2.62E-02 | 2.72E-01 |
|  | sphingomyelin (d18:1/20:2, d18:2/20:1, d16:1/22:2) | -3.21e-02 (-6.05e-02, -3.62e-03) | 3.00E-02 | 2.84E-01 |
| Dipeptide | threonylphenylalanine | -8.18e-02 (-1.39e-01, -2.44e-02) | 6.56E-03 | 1.32E-01 |
|  | glycylvaline | -1.42e-01 (-2.47e-01, -3.61e-02) | 1.02E-02 | 1.71E-01 |
|  | prolylproline | 1.19e-01 (2.75e-02, 2.1e-01) | 1.26E-02 | 2.01E-01 |
|  | cyclo(leu-pro) | 7.96e-02 (1.23e-02, 1.47e-01) | 2.30E-02 | 2.52E-01 |
| Urea cycle; Arginine and Proline Metabolism | homoarginine | -5.68e-02 (-9.58e-02, -1.77e-02) | 5.62E-03 | 1.25E-01 |
|  | ornithine | -3.12e-02 (-5.43e-02, -8.17e-03) | 9.57E-03 | 1.67E-01 |
|  | trans-4-hydroxyproline | -5.42e-02 (-9.6e-02, -1.24e-02) | 1.29E-02 | 2.03E-01 |
|  | N-acetylproline | 4.99e-02 (2.46e-03, 9.74e-02) | 4.25E-02 | 3.30E-01 |
| Fatty Acid, Dicarboxylate | hydroxy-CMPF | -9.95e-02 (-1.71e-01, -2.81e-02) | 7.75E-03 | 1.45E-01 |
|  | 3-carboxy-4-methyl-5-propyl-2-furanpropanoate (CMPF) | -9.68e-02 (-1.85e-01, -8.35e-03) | 3.50E-02 | 2.99E-01 |
|  | hexadecenedioate (C16:1-DC) | -5.61e-02 (-1.09e-01, -2.72e-03) | 4.27E-02 | 3.30E-01 |
| Glycine, Serine and Threonine Metabolism | serine | -3.95e-02 (-5.89e-02, -2.01e-02) | 1.43E-04 | 1.92E-02 |
|  | threonine | -4.59e-02 (-7.62e-02, -1.55e-02) | 4.04E-03 | 1.15E-01 |
|  | glycine | -2.41e-02 (-4.69e-02, -1.34e-03) | 4.12E-02 | 3.27E-01 |
| Hexosylceramides (HCER) | glycosyl ceramide (d18:1/23:1, d17:1/24:1) | -5.09e-02 (-8.39e-02, -1.79e-02) | 3.39E-03 | 1.15E-01 |
|  | glycosyl ceramide (d18:2/24:1, d18:1/24:2) | -3.73e-02 (-6.79e-02, -6.67e-03) | 1.93E-02 | 2.49E-01 |
|  | glycosyl-N-behenoyl-sphingadienine (d18:2/22:0) | -2.27e-02 (-4.2e-02, -3.51e-03) | 2.30E-02 | 2.52E-01 |
| Leucine, Isoleucine and Valine Metabolism | tigloylglycine | -7.34e-02 (-1.33e-01, -1.42e-02) | 1.74E-02 | 2.41E-01 |
|  | alpha-hydroxyisocaproate | -4.55e-02 (-8.31e-02, -7.96e-03) | 1.99E-02 | 2.49E-01 |
|  | leucine | -2.7e-02 (-5.28e-02, -1.2e-03) | 4.35E-02 | 3.32E-01 |
| Methionine, Cysteine, SAM and Taurine Metabolism | taurine | -4.46e-02 (-7.27e-02, -1.65e-02) | 2.58E-03 | 9.83E-02 |
|  | 3-sulfo-alanine | -5.43e-02 (-9.03e-02, -1.83e-02) | 4.13E-03 | 1.15E-01 |
|  | cysteine s-sulfate | -8.12e-02 (-1.41e-01, -2.12e-02) | 9.67E-03 | 1.67E-01 |
| Secondary Bile Acid Metabolism | taurocholenate sulfate | -1.02e-01 (-1.54e-01, -5e-02) | 2.43E-04 | 2.16E-02 |
|  | deoxycholate | -5.75e-02 (-1.04e-01, -1.14e-02) | 1.67E-02 | 2.35E-01 |
|  | lithocholate sulfate (1) | -1.4e-01 (-2.56e-01, -2.3e-02) | 2.14E-02 | 2.51E-01 |
| Alanine and Aspartate Metabolism | aspartate | -4.49e-02 (-8.21e-02, -7.59e-03) | 2.08E-02 | 2.51E-01 |
|  | asparagine | -2.85e-02 (-5.41e-02, -2.89e-03) | 3.21E-02 | 2.88E-01 |
| Ceramides | N-palmitoyl-heptadecasphingosine (d17:1/16:0) | -4.08e-02 (-7.15e-02, -1.01e-02) | 1.10E-02 | 1.77E-01 |
|  | N-palmitoyl-sphingadienine (d18:2/16:0) | -3.71e-02 (-6.89e-02, -5.28e-03) | 2.49E-02 | 2.66E-01 |
| Diacylglycerol | palmitoyl-linoleoyl-glycerol (16:0/18:2) [2] | -1.03e-01 (-1.76e-01, -3e-02) | 7.01E-03 | 1.39E-01 |
|  | linoleoyl-arachidonoyl-glycerol (18:2/20:4) [2] | -6.65e-02 (-1.26e-01, -6.41e-03) | 3.30E-02 | 2.94E-01 |
| Gamma-glutamyl Amino Acid | gamma-glutamylmethionine | -6.9e-02 (-1.17e-01, -2.11e-02) | 5.99E-03 | 1.25E-01 |
|  | gamma-glutamylthreonine | -4.58e-02 (-7.77e-02, -1.39e-02) | 6.15E-03 | 1.26E-01 |
| Glutamate Metabolism | glutamine | -2.04e-02 (-3.63e-02, -4.51e-03) | 1.39E-02 | 2.11E-01 |
| Glutamate Metabolism | 2-pyrrolidinone | 1.16e-01 (2.4e-02, 2.08e-01) | 1.55E-02 | 2.24E-01 |
| Medium Chain Fatty Acid | laurate (12:0) | 1.08e-01 (1.61e-02, 2.01e-01) | 2.39E-02 | 2.58E-01 |
|  | 10-undecenoate (11:1n1) | -5.64e-02 (-1.08e-01, -5.21e-03) | 3.38E-02 | 2.98E-01 |
| Phosphatidylinositol (PI) | 1-stearoyl-2-docosahexaenoyl-GPI (18:0/22:6) | -5.77e-02 (-9.43e-02, -2.11e-02) | 2.76E-03 | 1.02E-01 |
|  | 1-stearoyl-2-arachidonoyl-GPI (18:0/20:4) | -2.33e-02 (-3.87e-02, -7.89e-03) | 4.00E-03 | 1.15E-01 |
| Pyrimidine Metabolism, Uracil containing | 2'-deoxyuridine | -6.59e-02 (-9.41e-02, -3.77e-02) | 1.64E-05 | 4.38E-03 |
|  | N-acetyl-beta-alanine | 7.86e-02 (4.19e-02, 1.15e-01) | 6.85E-05 | 1.22E-02 |
| Tocopherol Metabolism | delta-CEHC | 9.37e-02 (1.7e-02, 1.7e-01) | 1.90E-02 | 2.49E-01 |
|  | gamma-CEHC | 1.01e-01 (1.14e-02, 1.91e-01) | 3.01E-02 | 2.84E-01 |
|  | indolepropionate | 7.89e-02 (6.87e-03, 1.51e-01) | 3.48E-02 | 2.99E-01 |
|  | indoleacetate | 5.87e-02 (3.8e-03, 1.14e-01) | 3.93E-02 | 3.23E-01 |
| Dihydrosphingomyelins | sphingomyelin (d18:0/18:0, d19:0/17:0) | -5.67e-02 (-1.12e-01, -1.53e-03) | 4.73E-02 | 3.51E-01 |
| Drug - Other | S-carboxymethyl-cysteine | -5.13e-02 (-9.8e-02, -4.65e-03) | 3.42E-02 | 2.98E-01 |
| Endocannabinoid | N-stearoyltaurine | -6.32e-02 (-1.05e-01, -2.11e-02) | 4.29E-03 | 1.15E-01 |
| Fatty Acid Metabolism (Acyl Carnitine, Hydroxy) | (S)-3-hydroxybutyrylcarnitine | -8.2e-02 (-1.55e-01, -9.1e-03) | 3.04E-02 | 2.84E-01 |
| Fatty Acid Metabolism (Acyl Carnitine, Long Chain Saturated) | margaroylcarnitine (C17) | -4.9e-02 (-9.38e-02, -4.15e-03) | 3.53E-02 | 2.99E-01 |
| Fatty Acid Metabolism (Acyl Carnitine, Polyunsaturated) | docosahexaenoylcarnitine (C22:6) | -1.55e-01 (-2.47e-01, -6.3e-02) | 1.45E-03 | 7.30E-02 |
| Fatty Acid Metabolism (Acyl Choline) | oleoylcholine | 5.54e-02 (9.9e-03, 1.01e-01) | 1.94E-02 | 2.49E-01 |
| Fatty Acid Metabolism (Acyl Glutamine) | 4-methylhexanoylglutamine | -9.6e-02 (-1.76e-01, -1.55e-02) | 2.19E-02 | 2.51E-01 |
| Fatty Acid, Amide | oleamide | -2.58e-01 (-4.21e-01, -9.6e-02) | 2.52E-03 | 9.83E-02 |
| Glutathione Metabolism | 5-oxoproline | -2.2e-02 (-4.04e-02, -3.53e-03) | 2.21E-02 | 2.51E-01 |
| Glycolysis, Gluconeogenesis, and Pyruvate Metabolism | lactate | -5.38e-02 (-8.07e-02, -2.69e-02) | 1.87E-04 | 2.16E-02 |
| Hemoglobin and Porphyrin Metabolism | biliverdin | -8.34e-02 (-1.31e-01, -3.6e-02) | 9.04E-04 | 5.68E-02 |
| Histidine Metabolism | 1-ribosyl-imidazoleacetate | 7.46e-02 (1.9e-02, 1.3e-01) | 1.02E-02 | 1.71E-01 |
| Lysine Metabolism | hydroxy-N6,N6,N6-trimethyllysine | 4.53e-02 (2.35e-03, 8.82e-02) | 4.19E-02 | 3.29E-01 |
| Lysoplasmalogen | 1-(1-enyl-oleoyl)-2-linoleoyl-GPE (P-18:1/18:2) | -5.74e-02 (-1.1e-01, -4.61e-03) | 3.62E-02 | 3.02E-01 |
| Modified Peptides | pyroglutamylglycine | -8.65e-02 (-1.48e-01, -2.48e-02) | 7.41E-03 | 1.41E-01 |
| Monoacylglycerol | 2-arachidonoylglycerol (20:4) | -6.22e-02 (-1.16e-01, -8.76e-03) | 2.52E-02 | 2.66E-01 |
| Oxidative Phosphorylation | phosphate | 6.11e-02 (2e-02, 1.02e-01) | 4.61E-03 | 1.20E-01 |
| Phospholipid Metabolism | glycerophosphoethanolamine | -2.31e-02 (-4.41e-02, -2.08e-03) | 3.43E-02 | 2.98E-01 |
| Plasmalogen | 1-stearyl-2-arachidonoyl-GPC (O-18:0/20:4) | -3.57e-02 (-6.75e-02, -3.96e-03) | 3.04E-02 | 2.84E-01 |
| Polyamine Metabolism | spermidine | -6.6e-02 (-1.12e-01, -2.03e-02) | 5.91E-03 | 1.25E-01 |
| Polypeptide | glu-gly-asn-val | -9.59e-02 (-1.86e-01, -5.39e-03) | 4.10E-02 | 3.27E-01 |
| Progestin Steroids | 5alpha-pregnan-3beta,20alpha-diol disulfate | -9.06e-02 (-1.78e-01, -2.91e-03) | 4.62E-02 | 3.48E-01 |
| Pyrimidine Metabolism, Cytidine containing | 3-methylcytidine | -5.58e-02 (-9.68e-02, -1.48e-02) | 9.26E-03 | 1.67E-01 |
| Pyrimidine Metabolism, Orotate containing | dihydroorotate | -4.06e-02 (-7.59e-02, -5.24e-03) | 2.72E-02 | 2.74E-01 |
| Pyrimidine Metabolism, Thymine containing | 3-aminoisobutyrate | -3.76e-02 (-7.07e-02, -4.5e-03) | 2.88E-02 | 2.84E-01 |
| TCA Cycle | fumarate | -5.33e-02 (-9.53e-02, -1.12e-02) | 1.52E-02 | 2.22E-01 |
| Tyrosine Metabolism | thyroxine | -4.46e-02 (-7.13e-02, -1.79e-02) | 1.56E-03 | 7.30E-02 |

1. Significantly changed metabolites ordered by metabolic pathways and P-value

2. The p-value was calculated using a linear mixed effect model with the metabolite level as the dependent variable and the duration from the baseline visit (measured in years) as the independent variable, adjusted for age at baseline, sex, and prior history of disease-modifying therapy.

**Supplementary Table 2**. Metabolite in magenta and green modules changed significantly following treatment.

|  |  | Linear mixed effect model results for individual metabolite change following treatment | | |
| --- | --- | --- | --- | --- |
| Module | Metabolite | MM^1^ | Estimate (95% CI) | P-value^2^ |
| Magenta | 2-palmitoyl-GPC (16:0) | 0.85 | -4.2e-02 (-7.61e-02, -7.82e-03) | 1.83E-02 |
|  | 2-stearoyl-GPE (18:0) | 0.82 | -3.04e-02 (-6.34e-02, 2.69e-03) | 7.55E-02 |
|  | 1-arachidonoyl-GPI (20:4) | 0.81 | -2.09e-02 (-4.9e-02, 7.12e-03) | 1.48E-01 |
|  | 2-stearoyl-GPI (18:0) | 0.79 | -1.33e-02 (-4.54e-02, 1.88e-02) | 4.20E-01 |
|  | 2-hydroxystearate | 0.78 | -3.06e-02 (-5.64e-02, -4.92e-03) | 2.21E-02 |
|  | 2-arachidonoyl-GPC (20:4) | 0.78 | -3.1e-02 (-6.89e-02, 6.98e-03) | 1.14E-01 |
|  | 1-stearoyl-GPI (18:0) | 0.78 | -2.06e-02 (-4.96e-02, 8.52e-03) | 1.70E-01 |
|  | 2-arachidonoyl-GPI (20:4) | 0.77 | -1.66e-02 (-4.43e-02, 1.12e-02) | 2.45E-01 |
|  | 2-hydroxypalmitate | 0.77 | -1.76e-02 (-4.08e-02, 5.65e-03) | 1.42E-01 |
|  | 1-stearoyl-GPC (18:0) | 0.75 | -1e-02 (-2.81e-02, 8.09e-03) | 2.82E-01 |
|  | N-stearoylserine | 0.75 | -3.55e-02 (-7.15e-02, 4.51e-04) | 5.65E-02 |
|  | 2-oleoyl-GPC (18:1) | 0.74 | -3.24e-02 (-6.58e-02, 1.08e-03) | 6.14E-02 |
|  | 1-palmitoyl-GPC (16:0) | 0.73 | -1.48e-02 (-3.11e-02, 1.51e-03) | 7.90E-02 |
|  | glycerophosphorylcholine (GPC) | 0.71 | -2.61e-02 (-5.38e-02, 1.61e-03) | 6.86E-02 |
|  | 1-stearoyl-2-arachidonoyl-GPI (18:0/20:4) | 0.70 | -2.33e-02 (-3.87e-02, -7.89e-03) | 4.00E-03 |
|  | 1-stearoyl-GPE (18:0) | 0.66 | -6.02e-03 (-3.26e-02, 2.05e-02) | 6.58E-01 |
|  | arachidonate (20:4n6) | 0.66 | -7.59e-03 (-3.99e-02, 2.48e-02) | 6.47E-01 |
|  | N-stearoyltaurine | 0.65 | -6.32e-02 (-1.05e-01, -2.11e-02) | 4.29E-03 |
|  | 1-nonadecanoyl-GPC (19:0) | 0.64 | -8.14e-03 (-4.07e-02, 2.44e-02) | 6.26E-01 |
|  | N-linoleoylserine | 0.62 | -2.26e-02 (-6.07e-02, 1.56e-02) | 2.50E-01 |
|  | 1-oleoyl-GPI (18:1) | 0.59 | -1.66e-02 (-5.49e-02, 2.16e-02) | 3.97E-01 |
|  | lysine | 0.58 | -2.12e-02 (-4.26e-02, 2.98e-04) | 5.68E-02 |
|  | N-oleoylserine | 0.57 | -1.56e-02 (-4.31e-02, 1.19e-02) | 2.70E-01 |
|  | 1-nonadecenoyl-GPC (19:1) | 0.54 | -2.54e-02 (-6.59e-02, 1.51e-02) | 2.23E-01 |
|  | 1-margaroyl-GPE (17:0) | 0.53 | -2.84e-02 (-7.44e-02, 1.76e-02) | 2.30E-01 |
|  | 1-arachidonoyl-GPE (20:4n6) | 0.52 | -9.9e-03 (-3.41e-02, 1.43e-02) | 4.25E-01 |
|  | 1-arachidonoyl-GPC (20:4n6) | 0.52 | -2.04e-03 (-2.74e-02, 2.33e-02) | 8.75E-01 |
|  | 1-palmitoyl-GPE (16:0) | 0.52 | -1.38e-02 (-4.23e-02, 1.47e-02) | 3.46E-01 |
|  | 1-linoleoyl-GPI (18:2) | 0.51 | -1.81e-02 (-5.27e-02, 1.65e-02) | 3.09E-01 |
|  | N-palmitoylserine | 0.45 | 1.47e-03 (-3.63e-02, 3.92e-02) | 9.39E-01 |
|  | spermidine | 0.44 | -6.6e-02 (-1.12e-01, -2.03e-02) | 5.91E-03 |
|  | glutamine | 0.43 | -2.04e-02 (-3.63e-02, -4.51e-03) | 1.39E-02 |
|  | 1-linoleoyl-GPG (18:2) | 0.39 | -2.33e-02 (-7.16e-02, 2.5e-02) | 3.48E-01 |
|  | arginine | 0.38 | 5.25e-03 (-1.96e-02, 3.01e-02) | 6.81E-01 |
|  | 2-arachidonoylglycerol (20:4) | 0.38 | -6.22e-02 (-1.16e-01, -8.76e-03) | 2.52E-02 |
|  | glycerol 3-phosphate | 0.38 | -1.2e-02 (-5.29e-02, 2.9e-02) | 5.68E-01 |
|  | 1-stearoyl-2-arachidonoyl-GPC (18:0/20:4) | 0.37 | -8.43e-03 (-1.97e-02, 2.87e-03) | 1.48E-01 |
|  | 2-linoleoylglycerol (18:2) | 0.36 | -4.47e-02 (-1.03e-01, 1.37e-02) | 1.38E-01 |
|  | S-carboxymethyl-cysteine | 0.36 | -5.13e-02 (-9.8e-02, -4.65e-03) | 3.42E-02 |
|  | carnitine | 0.36 | -4.3e-03 (-3.35e-02, 2.49e-02) | 7.74E-01 |
|  | 2-oleoylglycerol (18:1) | 0.35 | -1.41e-02 (-1.17e-01, 8.86e-02) | 7.88E-01 |
|  | 2-hydroxyarachidate | 0.34 | -7.35e-02 (-1.22e-01, -2.5e-02) | 3.94E-03 |
|  | succinate | 0.34 | -1.35e-02 (-3.49e-02, 7.82e-03) | 2.18E-01 |
|  | 5alpha-androstan-3beta,17alpha-diol disulfate | 0.33 | -1.25e-02 (-1.19e-01, 9.4e-02) | 8.19E-01 |
|  | adenine | 0.29 | -1.76e-02 (-4.64e-02, 1.12e-02) | 2.35E-01 |
|  | glutarate (C5-DC) | 0.28 | 1.65e-02 (-3.8e-02, 7.1e-02) | 5.55E-01 |
| Green | androstenediol (3beta,17beta) disulfate (2) | 0.93 | -7.13e-02 (-1.06e-01, -3.68e-02) | 1.15E-04 |
|  | 21-hydroxypregnenolone disulfate | 0.92 | -5.96e-02 (-9.15e-02, -2.78e-02) | 4.39E-04 |
|  | pregnenediol sulfate (C21H34O5S) | 0.92 | -5e-02 (-9.11e-02, -8.84e-03) | 1.96E-02 |
|  | dehydroepiandrosterone sulfate (DHEA-S) | 0.91 | -6.2e-02 (-1.05e-01, -1.93e-02) | 5.62E-03 |
|  | pregnenetriol sulfate | 0.91 | -5.59e-02 (-1.05e-01, -6.53e-03) | 2.93E-02 |
|  | pregnenolone sulfate | 0.89 | -7.68e-02 (-1.23e-01, -3.06e-02) | 1.64E-03 |
|  | pregnenediol disulfate (C21H34O8S2) | 0.87 | -9.61e-02 (-1.29e-01, -6.32e-02) | 1.78E-07 |
|  | androstenediol (3beta,17beta) disulfate (1) | 0.86 | -7.91e-02 (-1.2e-01, -3.76e-02) | 3.44E-04 |
|  | androstenediol (3beta,17beta) monosulfate (1) | 0.85 | -6.97e-02 (-1.2e-01, -1.9e-02) | 8.58E-03 |
|  | epiandrosterone sulfate | 0.84 | -6.84e-02 (-1.35e-01, -1.63e-03) | 4.80E-02 |
|  | androstenediol (3alpha, 17alpha) monosulfate (3) | 0.84 | -4.14e-02 (-8.87e-02, 5.86e-03) | 8.98E-02 |
|  | 5alpha-androstan-3beta,17beta-diol disulfate | 0.82 | -7.98e-02 (-1.48e-01, -1.11e-02) | 2.55E-02 |
|  | androsterone glucuronide | 0.82 | -5.62e-02 (-1.04e-01, -8.64e-03) | 2.31E-02 |
|  | pregnenetriol disulfate | 0.81 | -7.65e-02 (-1.12e-01, -4.14e-02) | 5.29E-05 |
|  | androsterone sulfate | 0.78 | -7.29e-02 (-1.41e-01, -4.36e-03) | 4.03E-02 |
|  | androstenediol (3alpha, 17alpha) monosulfate (2) | 0.76 | -6.42e-02 (-1.25e-01, -3.04e-03) | 4.29E-02 |
|  | 5alpha-androstan-3alpha,17beta-diol monosulfate (1) | 0.74 | -2.99e-02 (-9.29e-02, 3.31e-02) | 3.55E-01 |
|  | androstenediol (3beta,17beta) monosulfate (2) | 0.73 | -8.56e-02 (-1.39e-01, -3.24e-02) | 2.25E-03 |
|  | andro steroid monosulfate C19H28O6S (1) | 0.70 | -4.34e-02 (-1.14e-01, 2.75e-02) | 2.34E-01 |
|  | 16alpha-hydroxy DHEA 3-sulfate | 0.67 | -9.59e-02 (-1.52e-01, -3.97e-02) | 1.25E-03 |
|  | 5alpha-pregnan-3beta,20alpha-diol disulfate | 0.64 | -9.06e-02 (-1.78e-01, -2.91e-03) | 4.62E-02 |
|  | 5alpha-androstan-3beta,17beta-diol monosulfate (2) | 0.63 | 2.33e-02 (-9.32e-02, 1.4e-01) | 6.96E-01 |
|  | 5alpha-pregnan-3beta,20beta-diol monosulfate (1) | 0.61 | -2.64e-02 (-1.16e-01, 6.28e-02) | 5.64E-01 |
|  | 5alpha-pregnan-3beta,20alpha-diol monosulfate (2) | 0.59 | -4.69e-02 (-1.33e-01, 3.9e-02) | 2.88E-01 |
|  | 5alpha-androstan-3alpha,17beta-diol monosulfate (2) | 0.59 | -2.75e-02 (-9.26e-02, 3.77e-02) | 4.11E-01 |
|  | etiocholanolone glucuronide | 0.58 | -4.17e-02 (-8.59e-02, 2.55e-03) | 6.85E-02 |
|  | 17alpha-hydroxypregnanolone glucuronide | 0.55 | -9.33e-02 (-1.9e-01, 3.22e-03) | 6.18E-02 |
|  | glycocholenate sulfate | 0.54 | -3.32e-02 (-8.06e-02, 1.42e-02) | 1.74E-01 |
|  | biliverdin | 0.52 | -8.34e-02 (-1.31e-01, -3.6e-02) | 9.04E-04 |
|  | alpha-hydroxyisovalerate | 0.52 | -1.68e-02 (-7.09e-02, 3.72e-02) | 5.43E-01 |
|  | alpha-hydroxyisocaproate | 0.49 | -4.55e-02 (-8.31e-02, -7.96e-03) | 1.99E-02 |
|  | 2-hydroxy-3-methylvalerate | 0.49 | -1.2e-02 (-5.62e-02, 3.22e-02) | 5.97E-01 |
|  | bilirubin (E,E) | 0.47 | -3.59e-02 (-8.06e-02, 8.9e-03) | 1.20E-01 |
|  | 3beta,7alpha-dihydroxy-5-cholestenoate | 0.46 | -3.85e-02 (-8.87e-02, 1.16e-02) | 1.36E-01 |
|  | perfluorohexanesulfonate (PFHxS) | 0.46 | -1.15e-01 (-1.79e-01, -5.08e-02) | 7.29E-04 |
|  | 5-methyluridine (ribothymidine) | 0.45 | -1.03e-02 (-2.71e-02, 6.6e-03) | 2.37E-01 |
|  | pregnanediol-3-glucuronide | 0.45 | -6.96e-02 (-1.52e-01, 1.29e-02) | 1.02E-01 |
|  | taurocholenate sulfate | 0.45 | -1.02e-01 (-1.54e-01, -5e-02) | 2.43E-04 |
|  | metabolonic lactone sulfate | 0.45 | -5.03e-03 (-1e-01, 8.99e-02) | 9.18E-01 |
|  | 3beta-hydroxy-5-cholestenoate | 0.43 | -1.7e-02 (-5.84e-02, 2.44e-02) | 4.24E-01 |
|  | 6-bromotryptophan | 0.43 | -3.78e-02 (-7.64e-02, 7.23e-04) | 5.80E-02 |
|  | bilirubin (E,Z or Z,E) | 0.42 | -3e-02 (-6.85e-02, 8.47e-03) | 1.30E-01 |
|  | 17alpha-hydroxypregnenolone 3-sulfate | 0.42 | 1.61e-03 (-9.75e-02, 1.01e-01) | 9.75E-01 |
|  | 3-methylcytidine | 0.42 | -5.58e-02 (-9.68e-02, -1.48e-02) | 9.26E-03 |
|  | 11beta-hydroxyandrosterone glucuronide | 0.40 | 7.09e-03 (-4.11e-02, 5.53e-02) | 7.74E-01 |
|  | choline phosphate | 0.38 | -4.61e-03 (-3.54e-02, 2.62e-02) | 7.70E-01 |
|  | perfluorooctanoate (PFOA) | 0.38 | -1.11e-01 (-1.68e-01, -5.49e-02) | 2.20E-04 |
|  | bilirubin (Z,Z) | 0.38 | 3.83e-03 (-2.9e-02, 3.66e-02) | 8.20E-01 |
|  | pregnanolone/allopregnanolone sulfate | 0.37 | -8.64e-03 (-1.03e-01, 8.53e-02) | 8.57E-01 |
|  | 15-HETE | 0.36 | -1.71e-02 (-6.63e-02, 3.2e-02) | 4.96E-01 |
|  | N-acetylcarnosine | 0.36 | -2.47e-02 (-6.79e-02, 1.85e-02) | 2.66E-01 |
|  | cholesterol sulfate | 0.35 | 6.27e-03 (-1.09e-02, 2.35e-02) | 4.77E-01 |
|  | glycochenodeoxycholate 3-sulfate | 0.34 | -2.87e-02 (-1.01e-01, 4.32e-02) | 4.36E-01 |
|  | 12-HETE | 0.34 | 2.8e-03 (-8.49e-02, 9.05e-02) | 9.50E-01 |
|  | cortisone | 0.33 | -3.12e-02 (-7.5e-02, 1.26e-02) | 1.66E-01 |
|  | 5alpha-androstan-3alpha,17alpha-diol monosulfate | 0.33 | -5.93e-03 (-6.86e-02, 5.67e-02) | 8.53E-01 |
|  | urate | 0.32 | -1.64e-02 (-3.52e-02, 2.31e-03) | 8.96E-02 |
|  | glycylproline | 0.32 | 5.36e-02 (-7.13e-03, 1.14e-01) | 8.75E-02 |
|  | 21-hydroxypregnenolone monosulfate (1) | 0.31 | -2.02e-02 (-1.07e-01, 6.64e-02) | 6.50E-01 |
|  | 2-amino-4-cyanobutanoate | 0.30 | 4.9e-02 (5.6e-03, 9.25e-02) | 2.98E-02 |

1. MM: module-membership; MM for each metabolite is defined as the correlation of individual metabolites with the related eigen-metabolite.

2. The P-value is derived from using a linear mixed effect model with the metabolite level as the dependent variable and the duration from the baseline visit (measured in years) as the independent variable, adjusted for age at baseline, sex, and prior history of disease-modifying therapy.

**Supplementary Table 3.** Logistic regression results to assess the association between module eigenmetabolite change and odds of improvement based on ODRS

| Module | Odds Ratio for 1SD (95% CI)^1^ | P-value^2^ |
| --- | --- | --- |
| Magenta | 3.09e-01 (6.83e-02, 9.09e-01) | **3.15E-02** |
| Purple | 1.74e+00 (9.13e-01, 4.54e+00) | 9.76E-02 |
| Blue | 1.98e+00 (7.9e-01, 6.43e+00) | 1.51E-01 |
| Greenyellow | 1.36e+00 (6.84e-01, 3.01e+00) | 3.84E-01 |
| Tan | 5.96e-01 (1.41e-01, 2.19e+00) | 4.39E-01 |
| Black | 1.32e+00 (5.39e-01, 3.68e+00) | 5.46E-01 |
| Pink | 7.71e-01 (2.93e-01, 1.94e+00) | 5.76E-01 |
| Brown | 1.22e+00 (5.91e-01, 2.64e+00) | 5.85E-01 |
| Red | 8.22e-01 (2.77e-01, 2.42e+00) | 7.09E-01 |
| Turquoise | 8.92e-01 (4.54e-01, 1.76e+00) | 7.35E-01 |
| Yellow | 1.09e+00 (3.76e-01, 3.2e+00) | 8.70E-01 |
| Green | 1e+00 (1.64e-01, 6.35e+00) | 9.96E-01 |

1. SD: Standard deviation, CI: Confidence interval

2. P-value derived from logistic regression models on one SD change in module eigenmetabolite value adjusting for age at baseline, sex, years of follow-up, and DMT history

**Supplementary Table 4.** Logistic regression results to assess the association between metabolome change and odds of improvement based on ODRS, for metabolites with P-values < 0.05

| Metabolic Pathway | Metabolite | Odds Ratio for 1SD (95% CI)^1^ | P-value^2^ |
| --- | --- | --- | --- |
| Lysophospholipid | 2-arachidonoyl-GPC (20:4) | 1.45e-01 (5.1e-03, 6.15e-01) | 3.49E-03 |
|  | 1-stearoyl-GPI (18:0) | 1.65e-01 (2.02e-02, 6.91e-01) | 9.55E-03 |
|  | 2-palmitoyl-GPC (16:0) | 3.66e-01 (1.05e-01, 8.96e-01) | 2.65E-02 |
|  | 2-arachidonoyl-GPI (20:4) | 2.87e-01 (4.97e-02, 8.87e-01) | 2.72E-02 |
|  | 1-arachidonoyl-GPC (20:4n6) | 2.6e-01 (4.77e-02, 8.88e-01) | 3.01E-02 |
|  | 1-arachidonoyl-GPI (20:4) | 3.61e-01 (7.01e-02, 9.85e-01) | 4.62E-02 |
| Leucine, Isoleucine and Valine Metabolism | 3-hydroxyisobutyrate | 2.78e+00 (1.29e+00, 1.03e+01) | 5.61E-03 |
|  | N-acetylisoleucine | 2.35e+00 (1.13e+00, 7.37e+00) | 1.81E-02 |
|  | valine | 2.41e+00 (1.09e+00, 8.16e+00) | 2.57E-02 |
|  | alpha-hydroxyisocaproate | 2.46e+00 (1.06e+00, 9.48e+00) | 3.40E-02 |
|  | 3-methylglutaconate | 3.2e+00 (1.02e+00, 1.5e+01) | 4.54E-02 |
| Ascorbate and Aldarate Metabolism | oxalate (ethanedioate) | 2.52e-01 (2.54e-02, 7.06e-01) | 6.31E-03 |
|  | threonate | 3.52e-01 (8.89e-02, 9.15e-01) | 3.11E-02 |
|  | ascorbic acid 3-sulfate | 3.89e-01 (1.07e-01, 9.55e-01) | 3.85E-02 |
| Food Component/Plant | caffeic acid sulfate | 2.54e+00 (1.14e+00, 7.17e+00) | 2.11E-02 |
|  | alliin | 2.2e+00 (1.04e+00, 6.75e+00) | 3.71E-02 |
|  | trans-3,4-methyleneheptanoate | 5.46e-01 (2.5e-01, 9.94e-01) | 4.76E-02 |
| Histidine Metabolism | formiminoglutamate | 3.89e+00 (1.33e+00, 1.84e+01) | 6.48E-03 |
|  | 1-methylhistamine | 3.2e-01 (7.73e-02, 8.61e-01) | 2.19E-02 |
|  | imidazole lactate | 2.7e+00 (1.05e+00, 1.2e+01) | 3.75E-02 |
| Secondary Bile Acid Metabolism | glycodeoxycholate 3-sulfate | 8.93e+00 (1.83e+00, 1.18e+02) | 3.56E-03 |
|  | taurochenodeoxycholic acid 3-sulfate | 2.11e+00 (1.05e+00, 5.4e+00) | 3.64E-02 |
|  | taurolithocholate 3-sulfate | 2.09e+00 (1e+00, 6.22e+00) | 4.98E-02 |
| Fatty Acid, Dicarboxylate | maleate | 3.11e+00 (1.35e+00, 1.04e+01) | 5.55E-03 |
|  | docosadioate (C22-DC) | 3.44e-01 (1.14e-01, 8.09e-01) | 1.23E-02 |
| Fibrinogen Cleavage Peptide | fibrinopeptide A (7-16) | 2.02e+00 (1.02e+00, 5.59e+00) | 4.41E-02 |
|  | fibrinopeptide A (5-16) | 1.95e+00 (1.01e+00, 5.59e+00) | 4.69E-02 |
| Lactoyl Amino Acid | N-lactoyl phenylalanine | 3.27e+00 (1.22e+00, 1.59e+01) | 1.45E-02 |
|  | N-lactoyl leucine | 2.55e+00 (1.14e+00, 8.01e+00) | 1.99E-02 |
| Long Chain Polyunsaturated Fatty Acid (n3 and n6) | arachidonate (20:4n6) | 1.56e-01 (8.64e-03, 6.8e-01) | 5.74E-03 |
|  | dihomo-linolenate (20:3n3 or n6) | 2.21e-01 (3.01e-02, 7.05e-01) | 6.46E-03 |
| Lysine Metabolism | fructosyllysine | 2.69e+00 (1.16e+00, 8.66e+00) | 1.80E-02 |
|  | 6-oxopiperidine-2-carboxylate | 4.37e-01 (1.59e-01, 9.26e-01) | 2.88E-02 |
| Medium Chain Fatty Acid | heptanoate (7:0) | 3.55e+00 (1.39e+00, 1.39e+01) | 5.53E-03 |
|  | caprylate (8:0) | 2.84e+00 (1.04e+00, 1.24e+01) | 3.93E-02 |
| Monoacylglycerol | 2-linoleoylglycerol (18:2) | 3e-01 (7.34e-02, 7.24e-01) | 4.56E-03 |
|  | 2-arachidonoylglycerol (20:4) | 3.45e-01 (1e-01, 9.54e-01) | 4.01E-02 |
| Tyrosine Metabolism | N-acetyltyrosine | 2.76e+00 (1.14e+00, 8.77e+00) | 2.35E-02 |
|  | p-cresol glucuronide | 3.64e-01 (9.49e-02, 9.03e-01) | 2.69E-02 |
| Urea cycle; Arginine and Proline Metabolism | 2-oxoarginine | 3.15e+00 (1.16e+00, 1.56e+01) | 1.72E-02 |
|  | ornithine | 2.59e+00 (1.06e+00, 9.33e+00) | 3.49E-02 |
| Benzoate Metabolism | 4-hydroxyhippurate | 3.28e+00 (1.12e+00, 1.56e+01) | 2.74E-02 |
| Dipeptide | histidylalanine | 2.41e+00 (1.11e+00, 1.28e+01) | 2.04E-02 |
| Endocannabinoid | N-palmitoylserine | 4.35e-01 (1.61e-01, 8.87e-01) | 1.96E-02 |
| Fatty Acid Metabolism (Acyl Choline) | arachidonoylcholine | 1.86e-01 (1.71e-02, 7.67e-01) | 1.37E-02 |
| Glutamate Metabolism | glutamate | 2.7e+00 (1.02e+00, 8.97e+00) | 4.44E-02 |
| Guanidino and Acetamido Metabolism | 4-guanidinobutanoate | 2.22e+00 (1e+00, 7.43e+00) | 4.99E-02 |
| Long Chain Saturated Fatty Acid | behenate (22:0) | 2.48e-01 (4.77e-02, 7.64e-01) | 1.18E-02 |
| Modified Peptides | pyroglutamylvaline | 1.97e+00 (1.01e+00, 4.66e+00) | 4.81E-02 |
| Nicotinate and Nicotinamide Metabolism | quinolinate | 4.1e+00 (1.28e+00, 2.35e+01) | 1.41E-02 |
| Partially Characterized Molecules | pentose acid | 3.3e+00 (1.26e+00, 1.58e+01) | 8.93E-03 |
| Phenylalanine Metabolism | phenylalanine | 1.97e+00 (1.04e+00, 4.71e+00) | 3.71E-02 |
| Purine Metabolism, Guanine containing | N2-methylguanosine | 2.87e-01 (6.72e-02, 7.1e-01) | 4.06E-03 |
| Pyrimidine Metabolism, Cytidine containing | cytosine | 3.31e+00 (1.11e+00, 2.12e+01) | 2.51E-02 |
| Pyrimidine Metabolism, Orotate containing | orotidine | 3.18e+00 (1e+00, 1.46e+01) | 4.90E-02 |
| Sterol | 3beta,7alpha-dihydroxy-5-cholestenoate | 3.88e-01 (1.23e-01, 9.73e-01) | 4.31E-02 |

1. SD: Standard deviation, CI: Confidence interval

2. P-value derived from logistic regression models on one SD change in individual metabolite adjusting for age at baseline, sex, years of follow-up, and DMT history
